# Construction of Hierarchical Rod-Assembled Ce–Al–La–MOFs@PPy Composites for Highly Efficient Fluoride Removal from Water

**DOI:** 10.3390/nano16161015

**Published:** 2026-08-17

**Authors:** Xin Lin, Jiayi Tu, Jinyun Zhao, Fangfang Wu, Xingping Fu, Hao Lin, Jiapeng Hu

**Affiliations:** 1 Fujian Provincial Key Laboratory of Eco-Industrial Green Technology, Wuyi University, Wuyishan 354300, China; linxin820314@163.com (X.L.); tujiayi@163.com (J.T.); zhaojy@163.com (J.Z.); wyuwqhjp@163.com (J.H.); 2 Fujian Environmental Protection Design Institute Company Limited, Fuzhou 350001, China; 3 College of Resources and Environment, Fujian Agriculture and Forestry University, Fuzhou 350002, China

**Keywords:** rod-assembled, Ce–Al–La–MOFs@PPy, fluoride removal, solvothermal

## Abstract

In this study, a hierarchical rod-assembled, polypyrrole-modified Ce–Al–La trimetallic metal–organic framework (Ce–Al–La–MOFs@PPy) composite was successfully synthesized through a solvothermal method combined with in situ polymerization for efficient fluoride removal from aqueous solutions. The incorporation of conductive polypyrrole (PPy) into the multimetallic MOFs effectively enhanced the structural stability, surface properties, and adsorption performance of the material. Adsorption experiments demonstrated that the optimal metal-to-ligand molar ratio was 3:1, and the prepared adsorbent exhibited excellent fluoride removal performance under weakly acidic conditions (pH = 5). The maximum adsorption capacity calculated from the Langmuir model reached 216.92 mg·g^−1^ at 45 °C. Adsorption kinetics were well fitted by the pseudo-second-order model, indicating that the adsorption process was mainly governed by chemisorption. Mechanism investigations based on FTIR and XPS analyses demonstrated that fluoride removal mainly occurred through electrostatic attraction and coordination exchange between fluoride ions and the Ce/La active sites. In addition, the composite exhibited good selectivity, anti-interference ability toward coexisting ions, and satisfactory fluoride removal performance in practical industrial wastewater. These findings suggest that Ce–Al–La–MOFs@PPy is a promising adsorbent for efficient fluoride-contaminated wastewater treatment.

## 1. Introduction

As one of the most important freshwater resources worldwide, groundwater provides the majority of accessible freshwater for human use and plays an irreplaceable role in drinking water supply, agricultural irrigation, and industrial production. However, with the intensification of natural geological processes and human activities, fluoride ion (F^−^) contamination in groundwater has become increasingly severe, attracting widespread attention as a global environmental and public health issue [1,2]. Fluoride in groundwater mainly originates from the dissolution of fluoride-containing minerals such as fluorite, cryolite, and fluorapatite. In addition, industrial emissions from steel smelting and chemical industries, as well as the extensive use of fluoride-containing fertilizers and pesticides in agriculture, further aggravate fluoride accumulation in aquatic environments [3,4]. Due to its strong electronegativity and high chemical activity, fluoride exhibits considerable mobility in groundwater and can easily enter the human body through drinking water, leading to long-term accumulation. Fluoride exhibits a significant “dual effect” on human health. Appropriate fluoride intake (approximately 0.5–1.0 mg·L^−1^) can promote enamel calcification, enhance bone strength, and help prevent dental caries; therefore, fluoride is widely recognized as a vital trace element necessary for normal human growth and development. However, excessive long-term intake of fluoride can cause severe health problems, including dental fluorosis, skeletal fluorosis, bone deformities, neurological damage, thyroid dysfunction, and even Alzheimer’s disease [5,6,7]. Due to the potential health hazards caused by excessive fluoride exposure, the World Health Organization (WHO) has set the maximum allowable fluoride concentration in drinking water at 1.5 mg·L^−1^, whereas the Chinese Drinking Water Quality Standards require the fluoride level to be maintained below 1.0 mg·L^−1^.

At present, more than 50 countries and regions worldwide are affected by endemic fluorosis to varying degrees, and hundreds of millions of people are threatened by high-fluoride groundwater. Therefore, the development of efficient, stable, and highly selective defluoridation technologies is of great importance for ensuring drinking water safety, reducing fluoride pollution risks, and protecting human health. In recent years, adsorption technology has been regarded as one of the most promising methods for fluoride removal because of its simple operation, high treatment efficiency, low cost, and wide applicability [8,9,10]. Among various adsorbents, metal–organic frameworks (MOFs) and their composites have attracted considerable attention owing to their high specific surface area, abundant active sites, and tunable structures, showing great potential for efficient fluoride removal from water. For example, Song et al. [11] successfully synthesized rod-like Ce-MOFs adsorbents via a solvothermal method. Under pH = 4, the maximum adsorption capacity toward fluoride reached approximately 129.7 mg·g^−1^. The defluoridation process was mainly dominated by monolayer chemisorption accompanied by coordination interactions between Ce^3+^/Ce^4+^ and F^−^. Rashid et al. [12] synthesized a zirconium-based MOF-801 adsorbent via a solvothermal method and investigated its fluoride adsorption performance in aqueous solution. The adsorption process followed the pseudo-second-order kinetic model and was well described by the Langmuir isotherm model, with a maximum adsorption capacity of 19.42 mg·g^−1^. Moreover, MOF-801 retained approximately 79% fluoride removal efficiency after four regeneration cycles, indicating its potential as a recyclable Zr-MOF adsorbent for water defluoridation. Jeyaseelan and Viswanathan [13] synthesized tunable rare-earth-based MOFs using La^3+^ and Ce^3+^ as metal centers and BTC as the organic ligand, and the obtained La@BTC and Ce@BTC exhibited fluoride retention capacities of 4.985 and 4.930 mg F^−^ g^−1^, respectively. The fluoride adsorption was mainly attributed to electrostatic attraction and complexation between fluoride ions and La/Ce active sites. To further improve the adsorption capacity of single-metal MOFs, Hu et al. [14] synthesized bimetallic Ce–La-MOFs using a similar method. Under optimal conditions with a La:Ce:ATPA (Ligands) molar ratio of 3:1:2 and pH ≈ 3, the maximum Langmuir adsorption capacity reached 138.64 mg·g^−1^. The fluoride removal mechanism mainly involved synergistic electrostatic attraction and ion exchange. Moreover, the material exhibited excellent resistance to coexisting ion interference and good regeneration performance, maintaining approximately 73% fluoride removal efficiency after four regeneration cycles. Subsequently, the same research group further developed trimetallic MOFs adsorbents (Zr–La–Ce) with a broader applicable pH range [15]. Nevertheless, these defluoridation materials are still mainly based on metallic MOFs, which suffer from several limitations in practical applications, including poor electrical conductivity, particle agglomeration, insufficient structural stability, and limited anion transport efficiency [16,17]. These drawbacks can reduce active-site utilization and restrict adsorption kinetics. Therefore, integrating MOFs with conductive polymers to construct functional composite systems with synergistic effects has become an important strategy for further improving fluoride removal performance.

Polypyrrole (PPy) has attracted attention as a nitrogen-rich interfacial modifier for adsorbent materials because its molecular chains contain abundant pyrrolic nitrogen (–NH) groups. In aqueous media, these nitrogen sites can be protonated to generate positively charged domains, thereby favoring F^−^ uptake through electrostatic attraction [18,19]. The pyrrolic N–H groups may also contribute to fluoride binding through N–H···F interactions [20]. When incorporated onto the MOF surface, PPy introduces additional nitrogen-containing sites and modulates the local surface charge, which can facilitate the enrichment of fluoride at the adsorbent–solution interface. Liu et al. [21] successfully synthesized a core–shell structured UiO-66@PPy nanocomposite through in situ oxidative polymerization. The introduction of conductive PPy significantly enhanced fluoride adsorption performance, and the maximum Langmuir adsorption capacity reached 290.7 mg·g^−1^ at pH = 3. Fluoride removal by UiO-66@PPy mainly originated from coordination interactions between Zr active sites and F^−^, ion exchange, and electrostatic adsorption provided by PPy. Kang et al. [22] fabricated MOF/PPy composite electrodes via electrodeposition and applied them in hybrid capacitive deionization (HCDI) systems for fluoride removal. The incorporation of PPy markedly improved the conductivity and interfacial electron transfer capability of the MOF electrodes while increasing anion adsorption active sites, thereby effectively enhancing fluoride removal performance. Although Ce-MOFs, Ce–La-MOFs, and Zr–La–Ce MOFs have been reported for fluoride removal, most of these studies mainly focused on increasing the number of metal active sites within MOFs, while the regulation of interfacial charge transfer, anion transport, and polymer–MOF interfacial interactions has received limited attention.

Based on these considerations, a hierarchical rod-assembled polypyrrole-modified Ce–Al–La trimetallic metal–organic framework composite was constructed through solvothermal synthesis combined with in situ polymerization. Compared with previously reported single-metal and multimetallic MOFs, this composite integrates the strong fluoride affinity of Ce/La sites and the protonatable nitrogen-containing groups of PPy. This design is expected to enhance fluoride adsorption by improving active-site accessibility, reducing particle aggregation, and facilitating ion diffusion within the hierarchical structure. The fluoride adsorption performance of the composite was systematically evaluated under different metal-to-ligand molar ratios, solution pH values, and initial fluoride concentrations. In addition, SEM, XRD, FTIR, XPS, adsorption kinetics, and thermodynamic analyses were employed to clarify the relationship between structural characteristics and fluoride removal mechanisms, providing theoretical guidance for the design of high-performance multimetallic MOF-based adsorbents.

## 2. Materials and Methods

### 2.1. Experimental Materials

Lanthanum nitrate hexahydrate (La(NO_3_)_3_·6H_2_O, 99%) was purchased from Shandong Keyuan Biochemical Co., Ltd. (Shandong, China). Cerium nitrate hexahydrate (Ce(NO_3_)_3_·6H_2_O, 97%) was obtained from Shanghai Macklin Biochemical Co., Ltd. (Shanghai, China). Aluminum chloride hexahydrate (AlCl_3_·6H_2_O, AR), sodium fluoride (NaF, AR), absolute ethanol (AR), disodium hydrogen phosphate (Na_2_HPO_4_, AR), calcium chloride (CaCl_2_, AR), and magnesium chloride (MgCl_2_, AR) were supplied by Sinopharm Chemical Reagent Co., Ltd. (Shanghai, China). Pyrrole (CP) was obtained from Shanghai Aladdin Biochemical Technology Co., Ltd. (Shanghai, China). N,N-dimethylformamide (DMF, AR), 2-aminoterephthalic acid (98%), glacial acetic acid (99.5%), sodium chloride (AR), trisodium citrate dihydrate (AR), and sodium hydroxide (98%) were provided by Shandong Keyuan Biochemical Co., Ltd. (Jinan, Shandong, China). All reagents were directly used as received without any additional purification treatment.

### 2.2. Preparation of Ce–Al–La–MOFs@PPy

**Figure 1:**
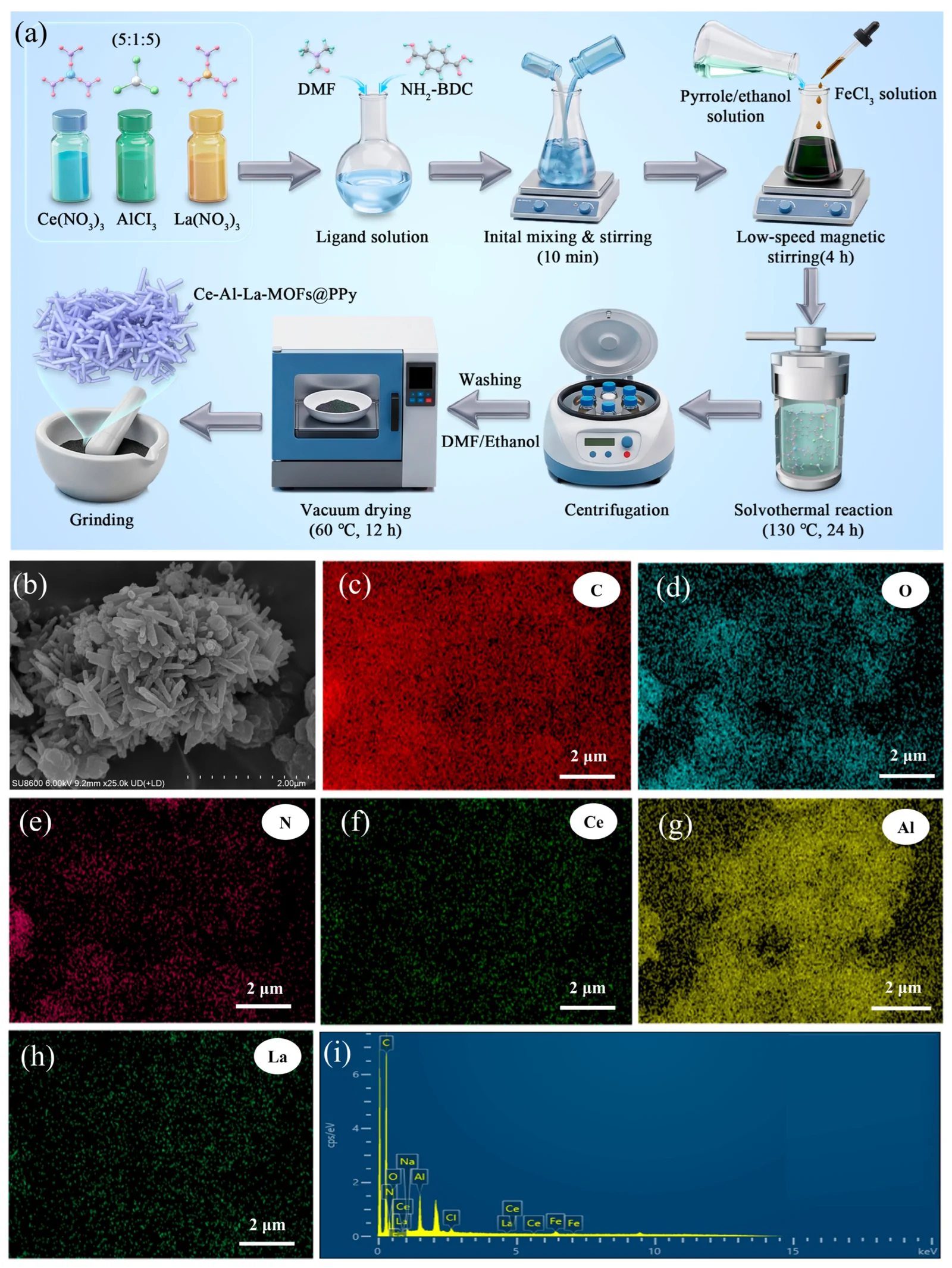
(**a**) Schematic illustration of the preparation procedure for Ce–Al–La–MOFs@PPy; (**b**) SEM image; (**c**–**h**) EDS elemental mapping images of C, O, N, Ce, Al, and La, respectively; (**i**) corresponding EDS spectrum of Ce–Al–La–MOFs@PPy.

The preparation process of Ce–Al–La–MOFs@PPy is illustrated in Figure 1. Ce(NO_3_)_3_·6H_2_O (2.17 g, 5 mmol), AlCl_3_·6H_2_O (0.241 g, 1 mmol), and La(NO_3_)_3_·6H_2_O (2.17 g, 5 mmol) were accurately weighed and dissolved in 40 mL of N,N-dimethylformamide (DMF), maintaining a Ce:Al:La molar ratio of 5:1:5. Subsequently, 2-aminoterephthalic acid (NH_2_-BDC, 0.665 g, 3.67 mmol) was dissolved in DMF, corresponding to a total metal-to-ligand molar ratio of 3:1. The two precursor solutions were mixed and magnetically stirred for 10 min to obtain a homogeneous solution. Thereafter, pyrrole monomer (6.94 mL, 0.10 mol) was dissolved in 30 mL of anhydrous ethanol and slowly introduced into the above mixture under low-speed magnetic stirring for 4 h. During this process, 20 mL of 0.5 mol·L^−1^ FeCl_3_ ethanol solution (10 mmol FeCl_3_) was added dropwise as the oxidizing agent to initiate the polymerization of pyrrole. The resulting suspension was transferred into a 100 mL Teflon-lined stainless-steel autoclave and heated at 130 °C for 24 h. After naturally cooling to room temperature, the obtained precipitate was collected by centrifugation, washed three times each with DMF and ethanol to remove unreacted species and residual Fe compounds, and then vacuum-dried at 60 °C for 12 h. Finally, the dried product was gently ground into a homogeneous powder and stored in a sealed vial for subsequent characterization and adsorption experiments.

To evaluate the influence of the molar ratio of metal ions to organic ligands on the adsorption properties of the prepared materials, a series of Ce–Al–La–MOFs@PPy samples were synthesized using cerium nitrate, aluminum chloride, and lanthanum nitrate as metal precursors while varying the dosage of the ligand NH_2_-BDC. Under the condition that the amounts of metal salts remained constant, the molar ratio of Ce:Al:La was fixed at 5:1:5. By adjusting the amount of ligand (NH_2_-BDC), the molar ratios of metal species to ligand were controlled at 4:1, 3:1, 2:1, 1:1, and 1:2, respectively, while all other synthesis conditions were kept unchanged. Thus, Ce–Al–La–MOFs@PPy materials with different compositions were obtained for subsequent comparative studies on adsorption performance.

### 2.3. Characterization of Ce–Al–La–MOFs@PPy

The morphology and elemental composition of the adsorbents were characterized using scanning electron microscopy coupled with energy-dispersive X-ray spectroscopy (SEM–EDX, Hitachi, Tokyo, Japan). The crystal structure of the adsorbents was analyzed by powder X-ray diffraction (XRD, Bruker D8 ADVANCE, Bruker, Billerica, MA, USA) with Cu Kα radiation over a scanning range of 5–70° at a scanning rate of 10° min^−1^. N_2_ adsorption–desorption isotherms were measured at −196 °C using a Micromeritics ASAP 2420 analyzer, and the specific surface area was calculated by the Brunauer–Emmett–Teller (BET) method. The thermal stability of the adsorbents was investigated using thermogravimetric analysis (TGA, DTA-60H, DTG, SHIMADZU, Kyoto, Japan) under a nitrogen atmosphere in the temperature range of 20–800 °C. Fourier transform infrared spectroscopy (FTIR, PerkinElmer, Yokohama, Japan) was employed to analyze the surface functional groups of the adsorbents within the wavenumber range of 400–4000 cm^−1^ using the KBr pellet method. The elemental composition and chemical valence states of the adsorbents were analyzed using X-ray photoelectron spectroscopy (XPS, ESCALAB 250, Thermo, Waltham, MA, USA) with a monochromatic Al *Kα* X-ray source (*hν* = 1486.6 eV). An appropriate amount of each sample was pressed into a pellet, mounted on the sample holder, and introduced into the XPS sample chamber. When the pressure in the sample chamber decreased below 2.0 × 10^−7^ mbar, the sample was transferred to the analysis chamber. The X-ray spot size was 400 μm, with an operating voltage of 12 kV and an emission current of 6 mA. Survey spectra were collected at a pass energy of 150 eV with an energy step of 1 eV, whereas high-resolution spectra were recorded at a pass energy of 50 eV with an energy step of 0.1 eV. The instrumental energy resolution was 0.5 eV, and the spatial resolution was 50 μm. The charging effect was compensated using the built-in charge neutralizer, and the C sp^2^ component was used as the reference peak for binding-energy calibration. After Shirley background subtraction, the high-resolution spectra were deconvoluted using a mixed Gaussian–Lorentzian line-shape function.

### 2.4. Adsorption Experiments

The fluoride concentration in aqueous solution was determined using a fluoride ion-selective electrode (ISE). A series of NaF standard solutions (0.1–100.0 mg·L^−1^) were prepared from a fluoride stock solution to establish the calibration curve prior to each set of measurements. The fluoride electrode exhibited excellent linearity within this concentration range (*R*^2^ > 0.999), and the method detection limit was approximately 0.02 mg·L^−1^. To eliminate the interference caused by ionic strength variation and metal ion complexation, an equal volume of Total Ionic Strength Adjustment Buffer (TISAB) was added to each sample before measurement. In a typical adsorption experiment, 50 mL of fluoride solution with the desired initial concentration was transferred into a 100 mL polyethylene centrifuge tube, followed by the addition of 0.010 g of adsorbent. Unless otherwise specified, the initial solution pH was adjusted using 0.1 mol L^−1^ HCl or 0.1 mol L^−1^ NaOH, and no additional background electrolyte was introduced to control the ionic strength. The suspension was shaken in a thermostatic water-bath shaker at 180 rpm for the predetermined contact time. Based on the adsorption kinetic study, an equilibrium time of 360 min was selected for all equilibrium adsorption experiments. After adsorption, the suspension was filtered through a 0.22 μm microporous membrane to remove the adsorbent particles. Subsequently, 10 mL of the filtrate was mixed with 10 mL of TISAB solution and diluted to 50 mL with deionized water. The resulting solution was transferred into a plastic beaker, and a fluoride ion-selective electrode together with a saturated potassium chloride reference electrode was immersed into the solution under gentle magnetic stirring. After the electrode potential became stable, the potential value was recorded. The equilibrium fluoride concentration was obtained from the calibration curve, and the adsorption capacity (*q_e_*) and fluoride removal efficiency (*η*) were calculated using the following equations [23,24]:

(1) $$q_{e}=\frac{V(C_{0}-C_{e})}{m},$$

(2) $$\eta =\frac{C_{0}-C_{e}}{C_{0}}\times 100\%,$$

The parameters are defined as follows: *q_e_* (mg·g^−1^) is the saturated adsorption capacity; *V* (L) represents the liquid phase volume; *C*_0_ and *C_e_* (mg·L^−1^) denote the initial and final concentrations of the fluoride solution, respectively; *m* (g) is the dosage of the adsorbent; and *η* (%) indicates the percentage removal.

Batch adsorption experiments were conducted to evaluate the effects of the metal-to-ligand molar ratio, initial fluoride concentration, solution pH, contact time, and temperature. Metal-to-ligand molar ratio experiments: Initial fluoride concentration = 30 mg·L^−1^; pH = 4; adsorbent dosage = 0.2 g L^−1^; temperature = 25 °C; contact time = 6 h. Initial fluoride concentration experiments: pH = 4; adsorbent dosage = 0.2 g L^−1^; temperature = 25 °C; contact time = 6 h. pH influence experiments: Initial fluoride concentration = 30 mg·L^−1^; adsorbent dosage = 0.2 g L^−1^; temperature = 25 °C; contact time = 6 h. Adsorption thermodynamics: pH = 5; adsorbent dosage = 0.2 g L^−1^; contact time = 6 h, temperatures were set at 25, 35, and 45 °C, with initial fluoride concentrations of 20, 25, 30, 35, 40, 50, and 60 mg·L^−1^. Adsorption kinetics: pH = 5; adsorbent dosage = 0.1 g L^−1^; temperature = 25 °C; initial fluoride concentrations = 10 and 20 mg·L^−1^. After adsorption, the supernatants were filtered through 0.45 μm membranes, and the residual fluoride concentrations were determined using an ion-selective electrode with TISAB. Notably, no additional dilution factor was applied during data processing; all concentrations were obtained directly from the calibration curve.

Additionally, we evaluated the interference of various background anions (Cl^−^, NO_3_^−^, SO_4_^2−^, CO_3_^2−^, HCO_3_^−^, and HPO_4_^2−^) present at concentrations between 10 and 80 mg·L^−1^. These experiments were conducted under the natural pH of the prepared solutions without any additional pH adjustment. The tests were performed at adsorbent dosage of 0.2 g L^−1^, contact time of 6 h, and 25 °C.

The pH_zpc_ of Ce–Al–La–MOFs@PPy was determined by the pH drift method. Briefly, 0.010 g of adsorbent was added to 50 mL of 0.01 mol L^−1^ NaCl solution with initial pH values ranging from 2.0 to 12.0. After shaking at 25 °C and 180 rpm for 24 h, the final pH was measured, and the pH_zpc_ was obtained from the intersection of the ΔpH curve with ΔpH = 0.

The fluoride-saturated adsorbent was regenerated by desorption using 0.1 mol L^−1^ NaOH solution. Typically, 0.010 g of the spent adsorbent was mixed with 50 mL of 0.1 mol L^−1^ NaOH (solid-to-liquid ratio of 0.2 g L^−1^) and shaken at 180 rpm for 12 h at room temperature to ensure complete desorption of fluoride ions. After desorption, the adsorbent was separated by centrifugation and thoroughly washed several times with deionized water until the filtrate reached approximately neutral pH (pH ≈ 7), thereby removing residual NaOH. The regenerated adsorbent was subsequently dried at 60 °C under vacuum for 12 h before being reused in the next adsorption cycle. The fluoride concentration in the desorption solution was determined using the fluoride ion-selective electrode following the same analytical procedure described above. The regenerated adsorbent was subjected to repeated adsorption–desorption cycles, and the fluoride removal efficiency and adsorption capacity were recorded to evaluate the regeneration performance and cyclic stability of Ce–Al–La–MOFs@PPy.

In the practical wastewater treatment experiments, 0.02 g of adsorbent was added to 50 mL of actual wastewater samples without any pretreatment or filtration. The adsorption experiments were conducted under room temperature with continuous shaking for 360 min. The initial pH of the wastewater was not adjusted, and the original pH conditions were directly maintained to evaluate the adsorption performance of the adsorbent in a realistic aquatic environment. After adsorption, the solid and liquid phases were separated, and the concentration of the target pollutants in the treated wastewater was determined.

## 3. Results and Discussion

### 3.1. Physicochemical Properties of Ce–Al–La–MOFs@PPy

#### 3.1.1. SEM Analysis

Figure 1b presents the SEM image of Ce–Al–La–MOFs@PPy. The material mainly consists of numerous short rod-like crystals assembled into flower-like cluster structures. Individual nanorods possess lengths ranging from several hundred nanometers to approximately 1 μm, while multiple nanorods are radially assembled into secondary particles with sizes of approximately 2–5 μm. This pronounced anisotropic growth and self-assembly behavior indicates that, under the synergistic coordination of multimetallic nodes and the interfacial regulation effect of PPy, the crystals preferentially grow into nanorod structures and further aggregate through surface-energy-driven assembly, thereby constructing a flower-like architecture with hierarchical pores, which is beneficial for ion diffusion and mass transfer in solution [25].

A uniform spatial distribution of C, O, and N is evident from the elemental mapping images, covering the entire individual particles. In particular, the homogeneous distribution of C and N confirms the successful incorporation of PPy, which is likely coated on the MOF surface or partially distributed within the porous framework. Meanwhile, Ce, Al, and La are uniformly dispersed throughout the particles without obvious aggregation, indicating the successful co-assembly of the three metal ions into a stable multimetallic MOF during the solvothermal process. The EDS spectrum further confirms the presence of C, O, N, Ce, Al, and La, demonstrating the successful construction of the Ce–Al–La–MOFs@PPy composite.

In addition, EDS detected a trace amount of Fe (approximately 0.2 wt%), which is attributed to residual Fe species originating from the FeCl_3_ oxidant used during the oxidative polymerization of pyrrole. Considering the extremely low Fe content and the repeated washing procedure employed after synthesis, most Fe species were effectively removed. Moreover, no distinguishable Fe 2p signal was detected in the XPS survey spectrum, which can be attributed to the different probing depths and detection sensitivities of EDS and XPS. EDS provides bulk compositional information on the micrometer scale, whereas XPS probes only the outermost surface (typically ~5–10 nm). Therefore, the trace residual Fe is unlikely to contribute significantly to the fluoride adsorption performance of the composite.

Overall, the nanorod-assembled flower-like morphology together with the homogeneous multielement distribution not only demonstrates the successful integration of PPy with Ce–Al–La–MOFs, but also suggests that the open hierarchical porous structure can significantly increase the exposure of metal active sites, thereby facilitating rapid fluoride diffusion and surface coordination-exchange reactions. The observed adsorption performance is intrinsically linked to these structural attributes, which underpin the material’s effectiveness.

#### 3.1.2. XRD and Thermogravimetric Analysis

**Figure 2:**
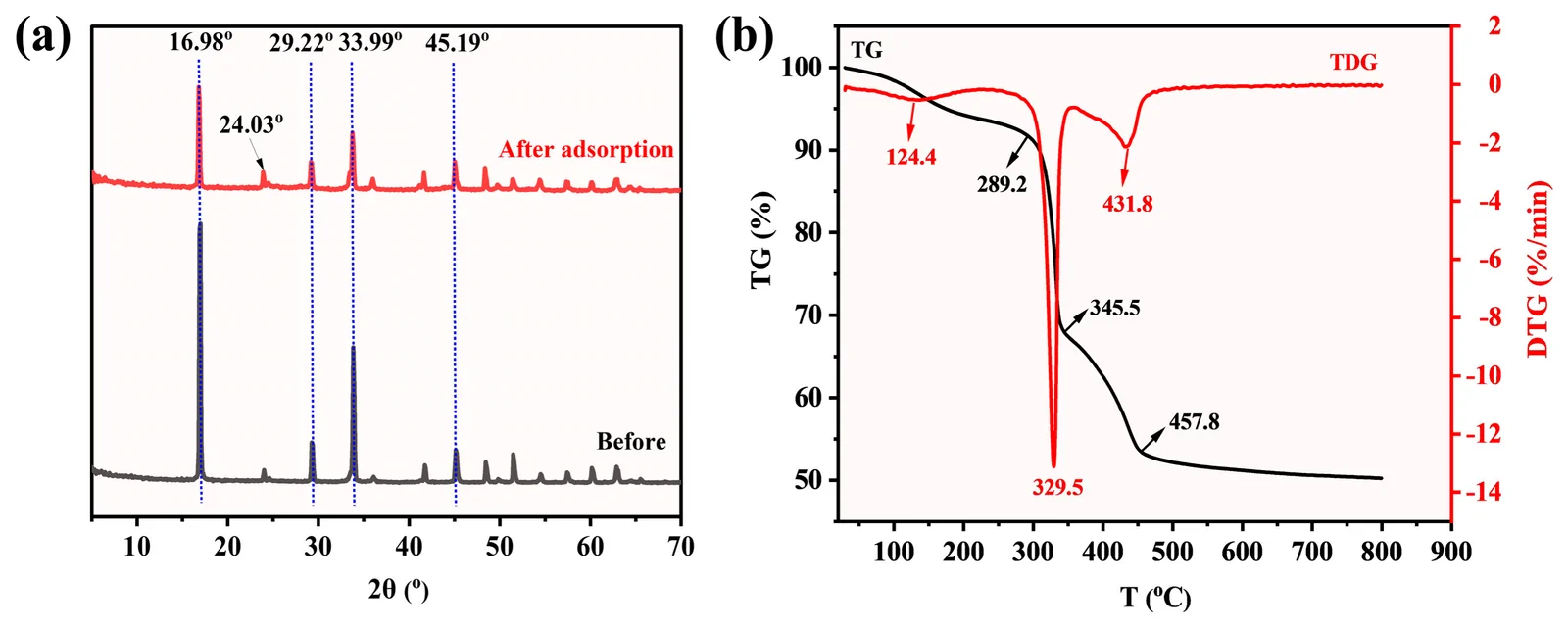
(**a**) XRD patterns and (**b**) TGA curves of Ce–Al–La–MOFs@PPy.

Figure 2a presents the XRD patterns of Ce–Al–La–MOFs@PPy before and after fluoride adsorption. The pristine composite exhibits diffraction peaks at 2θ values of 16.98°, 24.03°, 29.22°, 33.99°, 36.06°, 41.67°, 45.19°, 48.51°, 51.42°, 54.53°, 57.43°, 60.13°, and 63.04°, indicating the presence of crystalline or locally ordered domains within the metal-containing coordination composite. Three relatively intense diffraction peaks are observed at 16.98°, 29.22°, and 33.99°, consistent with the characteristic diffraction features of multimetal carboxylate coordination frameworks [26]. A broadened diffraction feature appears at 24.03°, which may originate from the semi-amorphous nature of PPy and may also be influenced by interactions between PPy and the MOF [27]. Collectively, these diffraction features support the successful formation of a crystalline multimetallic coordination framework in Ce–Al–La–MOFs@PPy and the incorporation of PPy into the composite system. After fluoride adsorption, most diffraction peaks became slightly weaker, suggesting that the local coordination environment and crystallinity of the framework were moderately altered during the adsorption process rather than indicating irreversible destruction of the framework. The decrease in diffraction intensity is mainly attributed to the coordination exchange between F^−^ ions and the surface hydroxyl groups of Ce/La active sites, accompanied by slight local structural rearrangement. These results indicate that fluoride adsorption primarily affects the local coordination structure while the overall crystalline framework of Ce–Al–La–MOFs@PPy remains largely preserved.

Figure 2b presents the thermogravimetric (TG) curve of Ce–Al–La–MOFs@PPy. The slight weight loss observed in the low-temperature region (<124.4 °C) is mainly attributed to the evaporation of adsorbed water and residual solvents within the pores. A significant weight loss occurs within the range of approximately 289.2–345.5 °C, corresponding to the decomposition of the organic ligand (NH_2_-BDC) and the initial degradation of PPy chains, accompanied by partial collapse of the MOFs. Further weight loss is observed within the range of approximately 345.5–457.8 °C, indicating complete decomposition and carbonization of the organic framework. Above 500 °C, the TG curve gradually becomes stable, with a final residual mass of 50.34 wt%, suggesting the formation of stable metal oxide phases such as CeO_2_, La_2_O_3_, and Al_2_O_3_. The relatively high decomposition temperature demonstrates that this multimetallic MOFs composite possesses excellent thermal stability.

#### 3.1.3. FTIR and Specific Surface Area Analysis

**Figure 3:**
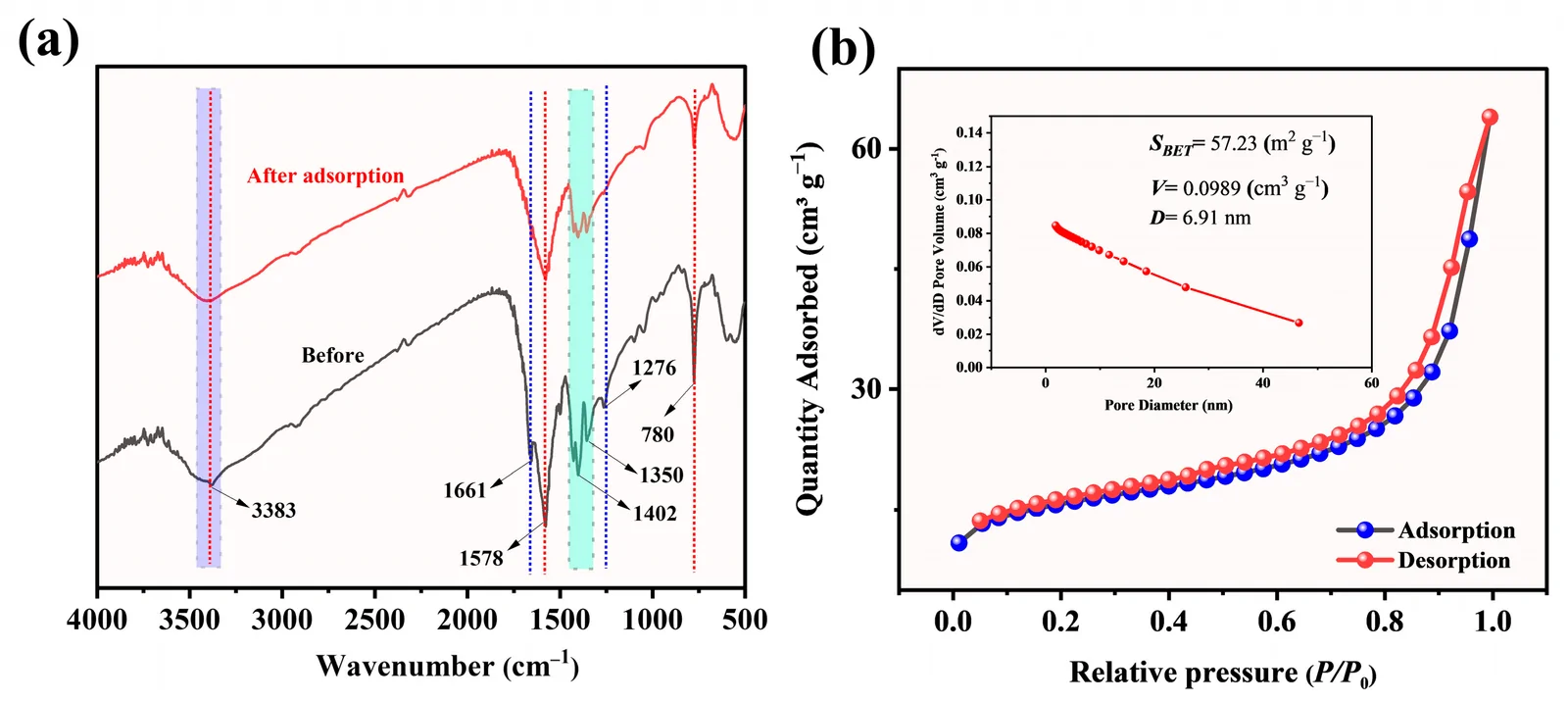
(**a**) FTIR spectra (The purple and cyan boxes highlight the key absorption peaks, while also enhancing the visual clarity of the spectral features.); (**b**) N_2_ adsorption/desorption isotherms for Ce–Al–La–MOFs@PPy, inset of BJH pore size distribution curve and textural parameters.

The FTIR analysis of Ce–Al–La–MOFs@PPy, conducted both before and after the adsorption process, is illustrated in Figure 3a. The spectra reveal three prominent peaks located at 3383, 1578, and 780 cm^−1^. The wide absorption band at 3383 cm^−1^ signifies the vibrational characteristics of hydroxyl groups originating from the hydration sphere of the MOFs [28].

Specifically, the absorption bands at 1578 cm^−1^ and 780 cm^−1^ are characteristic of the asymmetric stretching of –COO^−^ and the out-of-plane deformation of aromatic C–H, respectively. The symmetric stretching of –COO^−^ groups is evidenced by the peaks at 1402 and 1350 cm^−1^. Collectively, these spectral features verify that the NH_2_-BDC ligands have been effectively deprotonated and incorporated into the coordination network via bonding with Ce/Al/La metal nodes [29]. The absorption peak centered at 1661 cm^−1^, corresponding to the M–OH stretching mode, was completely eliminated following fluoride treatment. This observation strongly implies that the hydroxyl groups were replaced by fluoride ions via an ion-exchange mechanism [30]. A significant change in the FTIR spectrum was observed at 1276 cm^−1^ (C–N vibration of PPy), where the peak diminished after exposure to fluoride. This indicates that the process is chemically driven rather than purely physical. Specifically, F^−^ ions participate in coordination exchanges with metal nodes, modifying the vibrational state of the polymer.

Complementing this, the N_2_ isotherms in Figure 3b reveal a Type-IV behavior with an H3 hysteresis loop at high P/P_0_ values (0.7–1.0). Such features are diagnostic of mesoporous materials composed of loosely packed layered or rod-like nanostructures forming slit-like pores [31]. This observation is consistent with the rod-like and aggregated morphologies observed in the SEM images. The BET calculation results show that the material possesses a specific surface area of 57.30 m^2^ g^−1^, a total pore volume of 0.0989 cm^3^ g^−1^, and an average pore diameter of approximately 6.91 nm (inset of Figure 3b), which falls within the typical mesoporous range (2–50 nm). These results indicate that the internal pore structure is mainly composed of secondary pores formed between MOFs crystals together with pores generated by PPy coating or filling. The pore-size distribution curve reveals that the pore sizes are mainly concentrated within the range of approximately 3–20 nm, suggesting a relatively uniform pore distribution and well-developed hierarchical porous characteristics. Compared with conventional high-surface-area MOFs, the specific surface area of this material is relatively low, which may be attributed to the penetration of PPy into partial pore channels or coating on the crystal surfaces during the composite process. In addition, the presence of multimetallic nodes and crystal aggregation may partially block or shrink the pores, thereby reducing the overall surface area and pore volume. Nevertheless, the mesoporous-dominated structure is more favorable for the rapid diffusion and mass transfer of F^−^ ions in solution, while simultaneously exposing more metal coordination active sites and enhancing the efficiency of surface coordination-exchange reactions. Furthermore, the incorporation of PPy may form conductive or polarized networks on the material surface, which can further strengthen interfacial electronic interactions and ion adsorption capability. Therefore, the hierarchical mesoporous structure formed in Ce–Al–La–MOFs@PPy not only ensures good structural stability but also provides an important structural basis for its excellent fluoride adsorption performance.

### 3.2. Influence of Environmental Factors on Fluoride Uptake

#### 3.2.1. Effect of the Metal-to-Ligand Molar Ratio

**Figure 4:**
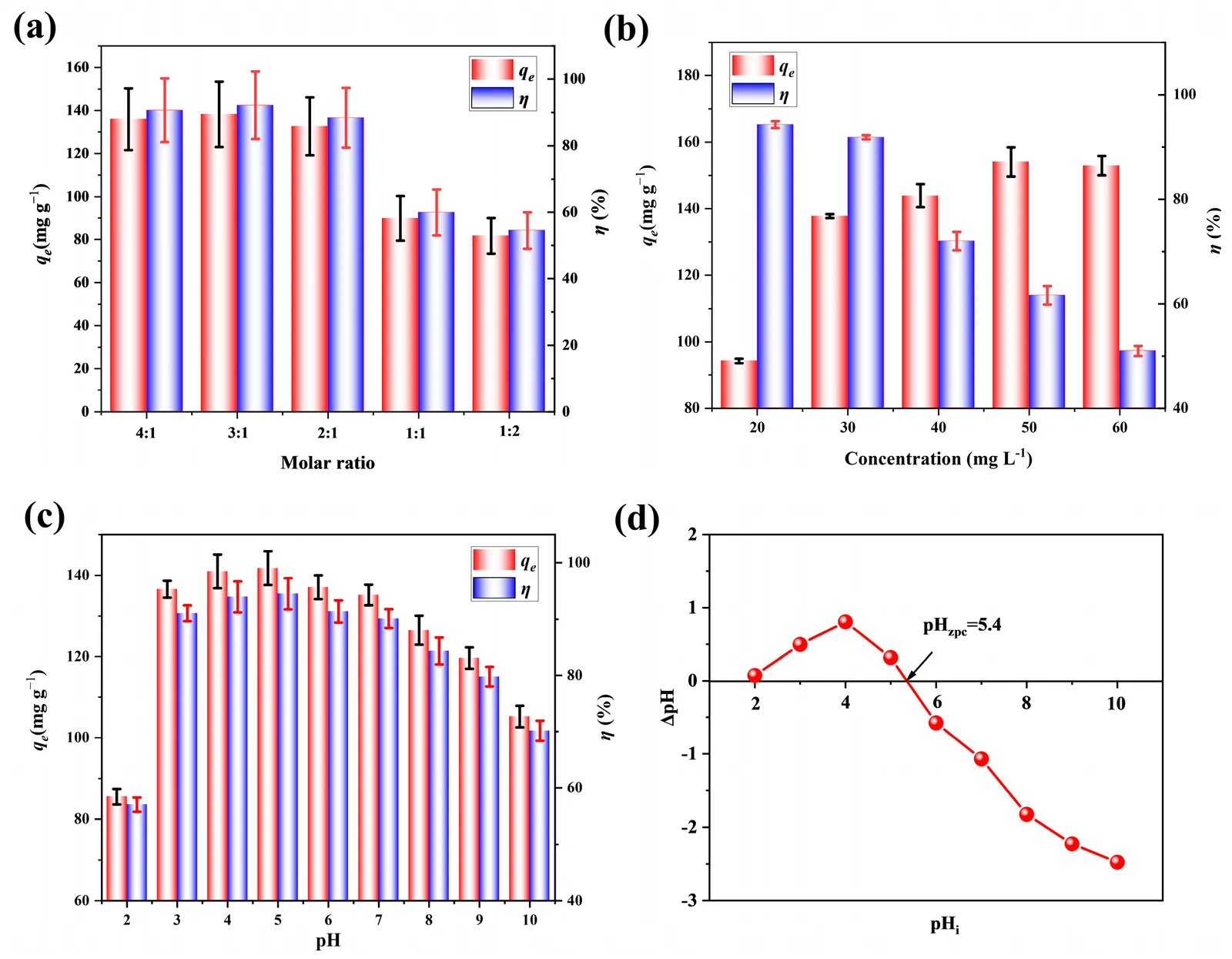
Effects on the F^−^ adsorption performance of Ce–Al–La–MOFs@PPy: (**a**) metal-to-ligand molar ratio; (**b**) initial F^−^ concentration; (**c**) effect of solution pH; (**d**) pHzpc curve.

The ratio between metal species and organic ligands plays an important role in determining the structure and performance of the materials. In this study, while maintaining a constant dosage of metal precursors, the amount of organic ligand (NH_2_-BDC) was systematically adjusted to vary the molar ratio between metal species and ligand, thereby investigating its influence on the material structure and adsorption behavior. Figure 4a illustrates the effect of the molar ratio between metal centers and organic ligands on the fluoride removal performance of Ce–Al–La–MOFs@PPy. Both the adsorption capacity and removal rate peaked at an optimal ligand dosage, beyond which they declined progressively. When the molar ratio of metal species to ligand was 3:1, Ce–Al–La–MOFs@PPy exhibited the best adsorption performance, with an adsorption capacity of 138.25 mg·g^−1^ and a fluoride removal efficiency of 92.16%. At relatively low ligand dosages (e.g., 4:1), insufficient coordination prevented the complete formation of the MOF structure, thereby limiting the effective utilization of active sites [32]. In contrast, at relatively high ligand dosages (e.g., 1:1 and 1:2), excessive organic ligands caused pore blockage and dilution of metal active sites, leading to reduced adsorption performance [33]. Therefore, a molar ratio of 3:1 between metal species and 2-aminoterephthalic acid was selected in this study, and all subsequent Ce–Al–La–MOFs@PPy samples were synthesized using this optimized ratio.

#### 3.2.2. Effect of Initial Fluoride Concentration

The relationship between initial fluoride levels and adsorption performance is illustrated in Figure 4b. An increase in initial concentration resulted in a gradual enhancement of adsorption capacity (from 94.23 to 154.09 mg·g^−1^) but a reduction in removal efficiency (from 94.24% to 50.99%). This discrepancy arises because a higher concentration gradient accelerates the diffusion of F^−^ ions towards the adsorbent. However, since the number of binding sites on Ce–Al–La–MOFs@PPy is limited, they become saturated at higher concentrations (60 mg/L). Consequently, although the total amount adsorbed increases, it constitutes a smaller fraction of the total fluoride in the solution, explaining the decrease in removal efficiency. Comparable observations are prevalent in the literature regarding various adsorbents [34].

#### 3.2.3. Effect of pH

The fluoride adsorption mechanism is elucidated by combining pH-variation studies with pHzpc determination. The pHzpc of 5.4 (Figure 4d) indicates that in environments with a pH lower than this threshold, the adsorbent surface becomes positively polarized via protonation, thereby promoting the sequestration of fluoride anions. This behavior is consistent with the typical characteristic of porous adsorbents exhibiting positively charged surfaces below their pH_zpc_ values [35,36]. In contrast, when the pH exceeds 5.4, the material surface gradually undergoes deprotonation and becomes negatively charged, resulting in electrostatic repulsion toward F^−^ ions and thus hindering the adsorption process. Experimental results show that as the solution pH increased from 2 to 5, both the adsorption capacity and removal efficiency continuously increased, reaching maximum values of 141.75 mg·g^−1^ and 94.50%, respectively, at pH = 5 (Figure 4c). This indicates that under weakly acidic conditions, the adsorbent surface still maintains strong positive charges, which effectively attract F^−^ ions. Meanwhile, coordination exchange interactions between F^−^ and metal active sites such as Ce, La, and Al are also more favorable under these conditions. Therefore, both electrostatic attraction and surface coordination synergistically promote the defluoridation process. However, under strongly acidic conditions (pH = 2), although the material surface carries more positive charges, the excessive H^+^ concentration competes with F^−^ for adsorption sites. In addition, part of the fluoride may combine with H^+^ to form HF, thereby reducing the concentration of free F^−^ ions in solution and leading to relatively lower adsorption capacity and removal efficiency. As the pH further increases to around 5, the competitive effect of H^+^ significantly decreases, while the material surface still remains below the pH_zpc_, thus retaining strong electrostatic attraction and high active-site utilization efficiency, resulting in optimal adsorption performance. When the pH further increases to 6–10, the system gradually exceeds the pH_zpc_ value, and the surface of the material changes from positively charged to negatively charged. Consequently, the electrostatic attraction between F^−^ and Ce–Al–La–MOFs@PPy weakens or even transforms into electrostatic repulsion. Simultaneously, the increasing concentration of OH^−^ ions competes with F^−^ for coordination sites on Ce, La, and other metal centers, thereby suppressing the binding and immobilization of fluoride ions. Concurrently, the abundance of OH^−^ ions at higher pH values leads to competitive occupation of the Ce/La metal centers. This steric and electronic competition effectively impedes the coordination and subsequent immobilization of fluoride anions. As a result, both the *q_e_* and *η* gradually decrease. Overall, the adsorption of F^−^ by Ce–Al–La–MOFs@PPy is jointly influenced by surface charge regulation, H^+^/OH^−^ competitive effects, and coordination exchange interactions with metal active sites. Weakly acidic conditions with pH values close to but slightly lower than the pH_zpc_ are the most favorable for achieving the optimal defluoridation performance of Ce–Al–La–MOFs@PPy.

#### 3.2.4. Fluoride Adsorption Behavior of PPy, Ce–Al–La–MOFs, and Ce–Al–La–MOFs@PPy

To preliminarily evaluate the contribution of PPy to the fluoride adsorption performance, the adsorption capacities of pure PPy, Ce–Al–La–MOFs, and Ce–Al–La–MOFs@PPy were compared under identical experimental conditions (Figure S1). As shown in Figure S1, all three adsorbents exhibited a rapid adsorption stage within the initial 60 min, followed by a gradual approach to adsorption equilibrium. This behavior suggests that abundant vacant active sites were initially available on the adsorbent surface, leading to fast fluoride uptake, whereas the adsorption rate gradually decreased as the active sites became progressively occupied. Among the three materials, pure PPy showed the lowest adsorption capacity, reaching an equilibrium value of only approximately 49.12 mg·g^−1^, indicating that the nitrogen-containing functional groups in PPy contribute only limited fluoride adsorption through electrostatic interaction and weak surface complexation. In contrast, Ce–Al–La–MOFs exhibited a significantly higher equilibrium adsorption capacity of approximately 121.55 mg·g^−1^. After introducing PPy into the MOF, the equilibrium adsorption capacity further increased to approximately 133.49 mg·g^−1^, representing an improvement of nearly 10% compared with the pristine Ce–Al–La–MOFs.

The enhanced adsorption performance of Ce–Al–La–MOFs@PPy can be attributed to the synergistic effect between the conductive PPy network and the multimetallic MOF. On the one hand, PPy improves the dispersion of MOF particles and suppresses their aggregation, thereby exposing more accessible adsorption sites. On the other hand, the nitrogen-containing functional groups in PPy facilitate the enrichment of fluoride ions near the adsorbent surface through electrostatic interaction, promoting subsequent coordination exchange between fluoride ions and the Ce/La active centers. Therefore, although PPy itself exhibits relatively limited fluoride adsorption capacity, its incorporation effectively enhances the utilization efficiency of the active metal sites, resulting in faster adsorption kinetics and a higher equilibrium adsorption capacity for the composite.

### 3.3. Adsorption Thermodynamics

**Figure 5:**
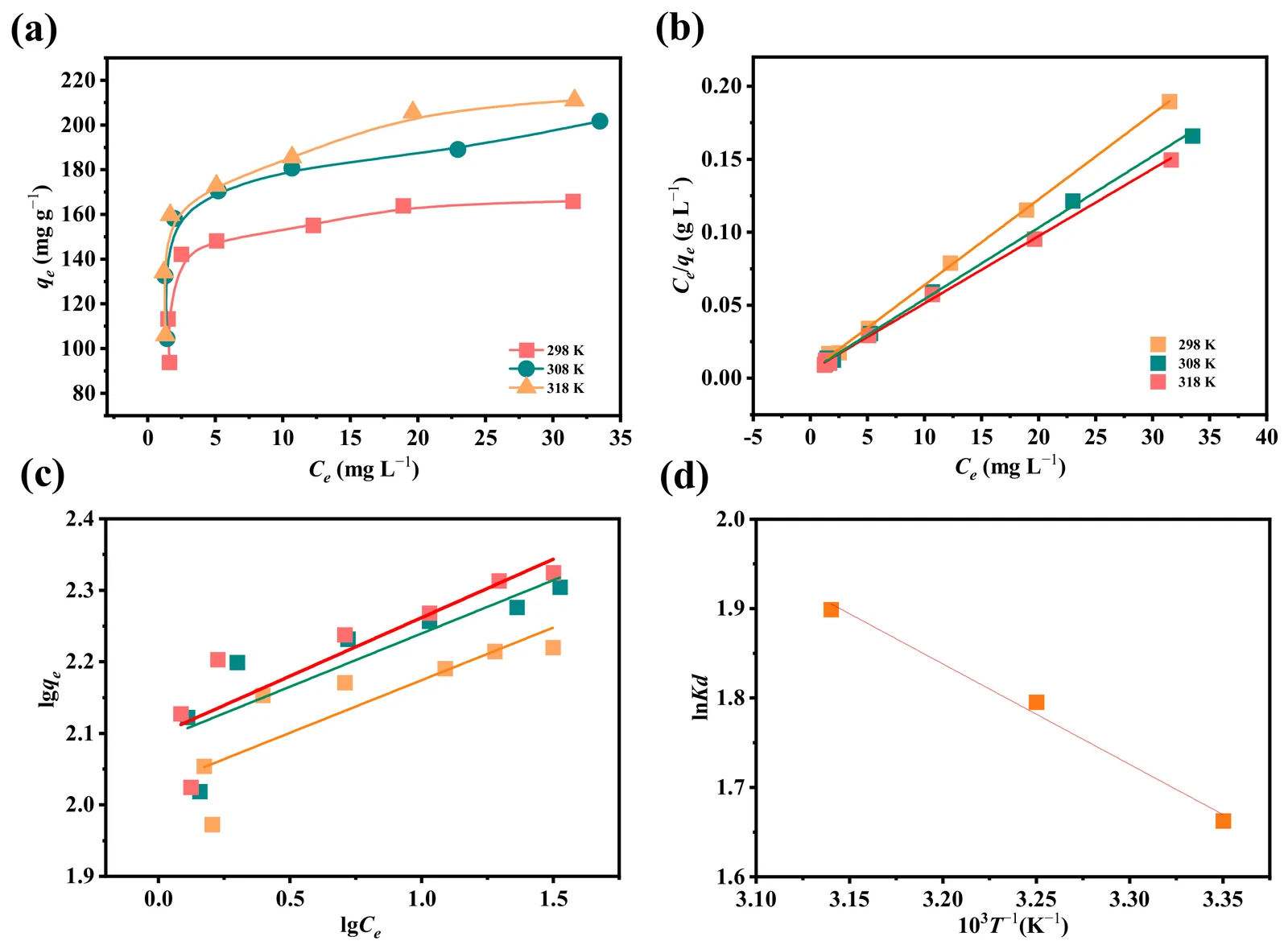
(**a**) Adsorption thermodynamics curves; (**b**) Langmuir model; (**c**) Freundlich model; (**d**) linear relationship between ln*K_d_* and 10^3^/*T*.

To gain insights into the enthalpic and entropic aspects of adsorption, the isotherm data were correlated with the Langmuir and Freundlich models. The adsorption isotherms (Figure 5) display a hyperbolic increase in *q_e_* with concentration, eventually reaching a maximum uptake. This saturation kinetics suggests that at low concentrations, the adsorbent surface is highly accessible, but becomes congested with fluoride ions at higher loadings, limiting further adsorption [15]. Temperature played a pivotal role in promoting adsorption, as evidenced by the highest *q_e_* observed at 45 °C, followed by 35 °C and 25 °C. Model fitting analysis revealed that the Langmuir isotherm provided a superior correlation (*R*^2^ > 0.998) compared to the Freundlich model (Table 1). This statistical dominance implies a uniform distribution of active sites and a monolayer adsorption behavior governing the interaction between F^−^ and Ce–Al–La–MOFs@PPy [14,37]. Accordingly, the calculated *Q_m_* creased from 170.36 to 216.92 mg·g^−1^ with rising temperature, indicating that the process is thermodynamically favorable at elevated temperatures (Table 1). The thermodynamic feasibility of the adsorption was evaluated by calculating enthalpy change (Δ*H*°), entropy change (Δ*S*°), and Gibbs free energy change (Δ*G*°) based on the temperature-dependent distribution coefficient (*K_d_*) (Text S1). As presented in Table 2 and Figure 5d, the negative Δ*G*° values signify a spontaneous and thermodynamically favorable process. The increase in the absolute value of Δ*G*° with temperature implies that the system becomes more spontaneous under thermal activation. The positive Δ*H*° (9.3330 kJ·mol^−1^) confirms the endothermic nature of the interaction, where heat absorption facilitates the uptake of F^−^.

Aligning with the XPS findings, Δ*S*° value (45.1522 J·mol^−1^·K^−1^) reflects a greater degree of freedom at the interface upon adsorption. This phenomenon is attributed to the displacement of water molecules from the hydration shells of F^−^ ions and the adsorbent surface. The liberation of these previously constrained water molecules and the reconfiguration of surface hydroxyls lead to a net increase in randomness within the system [38]. In summary, the adsorption of F^−^ by Ce–Al–La–MOFs@PPy is characterized by spontaneity, endothermic behavior, and entropy increase.

### 3.4. Adsorption Kinetics

**Figure 6:**
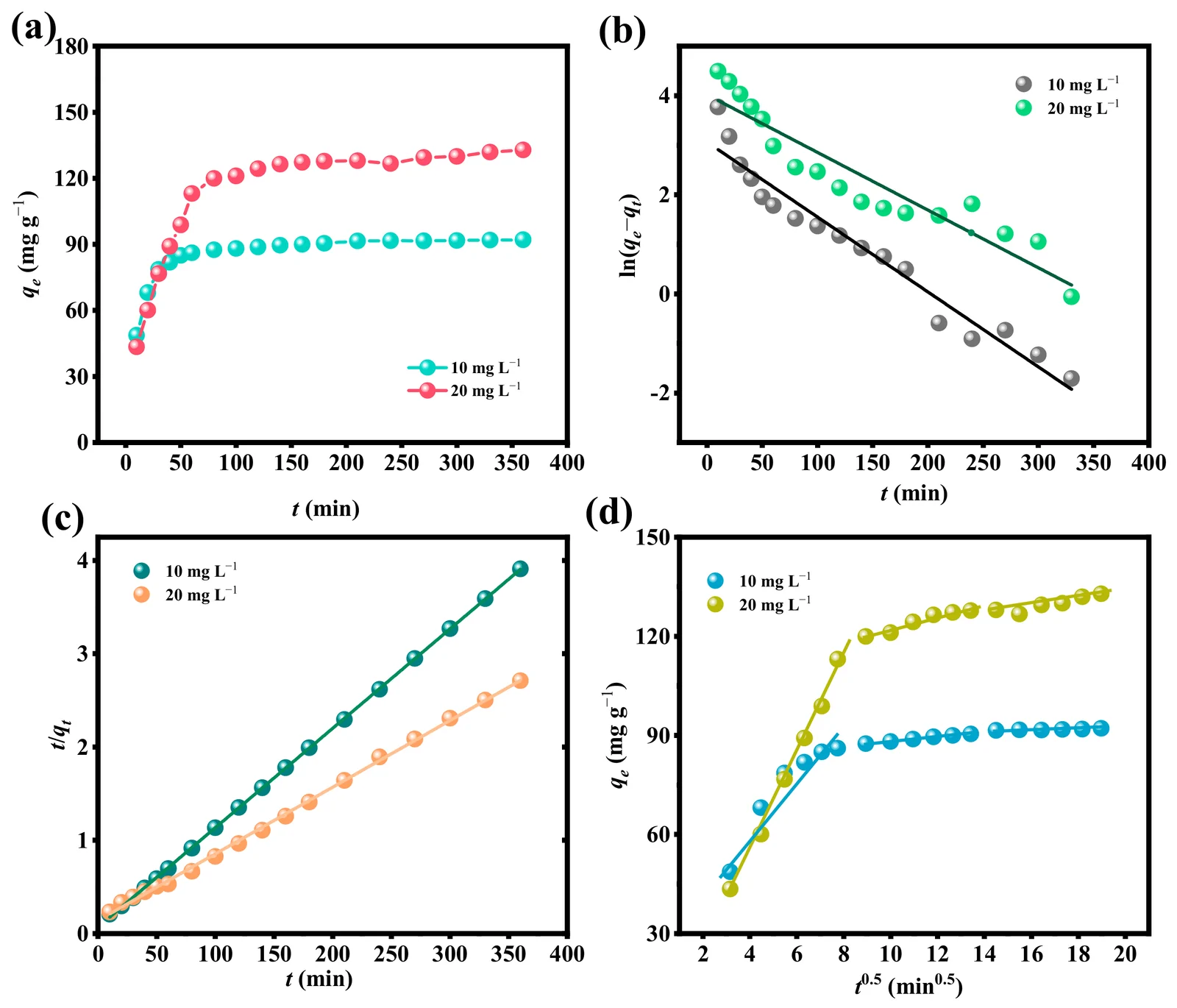
Adsorption kinetics of fluoride ions on Ce–Al–La–MOFs@PPy: (**a**) adsorption kinetic curves; (**b**) PFO kinetic model; (**c**) PSO model; (**d**) intraparticle diffusion model.

Figure 6a presents the adsorption kinetics of F^−^ onto Ce–Al–La–MOFs@PPy. The adsorption process exhibits a distinct two-stage characteristic. In the early stages of adsorption (0–50 min), a sharp increase in uptake was observed. This is attributed to the high density of accessible binding sites and the maximal driving force provided by the concentration gradient, which promotes the swift migration of fluoride ions to the adsorbent interface. Subsequently, the system enters a slower adsorption stage, during which the adsorption rate gradually decreases and eventually approaches equilibrium. At this stage, the effective surface active sites become progressively occupied, diffusion resistance increases, and the adsorption system gradually reaches equilibrium. In comparison, the equilibrium adsorption capacity obtained at a higher initial concentration (20 mg·L^−1^) is significantly greater than that at 10 mg·L^−1^, indicating that increasing fluoride concentration enhances the mass-transfer driving force and thereby improves adsorption capacity. To further characterize the kinetic behavior of the adsorption process, the experimental data were fitted using pseudo-first-order, pseudo-second-order, and intraparticle diffusion models. The detailed calculation methods for these models are provided in Supporting Information Text S2. Figure 6b–d illustrates the regression results for the pseudo-first-order (PFO), pseudo-second-order (PSO), and intraparticle diffusion models, and the associated kinetic parameters are summarized in Table 3. Statistical evaluation indicates that the adsorption kinetics are more accurately described by the PSO model, which demonstrates a higher coefficient of determination (*R*^2^) than the PFO model.

Under fluoride concentrations of 10 mg·L^−1^ and 20 mg·L^−1^, the correlation coefficients (R^2^) of the PSO model are 0.9999 and 0.9996, respectively, which are significantly higher than those of the PSD model (0.9020 and 0.9308, respectively). These results indicate that the adsorption rate of F^−^ onto Ce–Al–La–MOFs@PPy is mainly controlled by surface reaction processes rather than simple physical diffusion. Meanwhile, the equilibrium adsorption capacities calculated from the PSO model are 93.0233 and 136.7989 mg·g^−1^, respectively, which are consistent with the experimental trends, further demonstrating that this model can more accurately describe the adsorption behavior. PSO kinetics generally imply the involvement of electron exchange or chemical interactions during adsorption, such as coordination interactions or complexation. Therefore, it can be inferred that coordination exchange or electrostatic adsorption may occur between F^−^ ions and Ce or La metal active sites, thereby governing the adsorption rate. The fitting results of the intraparticle diffusion model show that the adsorption curves exhibit multilinear characteristics and do not pass through the origin, indicating that intraparticle diffusion is not the sole rate-controlling step for F^−^ adsorption onto Ce–Al–La–MOFs@PPy. Instead, the overall adsorption rate is jointly controlled by intraparticle diffusion, liquid-film diffusion, and surface reaction processes [39]. The relatively low *R*^2^ values observed in Stage III are mainly attributed to the fact that adsorption had nearly reached equilibrium, where the variation in *q_t_* with *t*^0.5^ became very small (Table 4). Therefore, the Stage III fitting is only used to indicate the equilibrium tendency rather than to extract a reliable diffusion rate constant. Therefore, based on the above kinetic analyses, it can be concluded that the adsorption of F^−^ onto Ce–Al–La–MOFs@PPy is governed by a multistep synergistic process. The PSO kinetic model best describes the adsorption system, suggesting that the metal active sites on the surface of Ce–Al–La–MOFs@PPy play a key role during adsorption and may promote efficient fluoride removal through the combined effects of electrostatic attraction and coordination interactions.

### 3.5. Effect of Coexisting Ions

**Figure 7:**
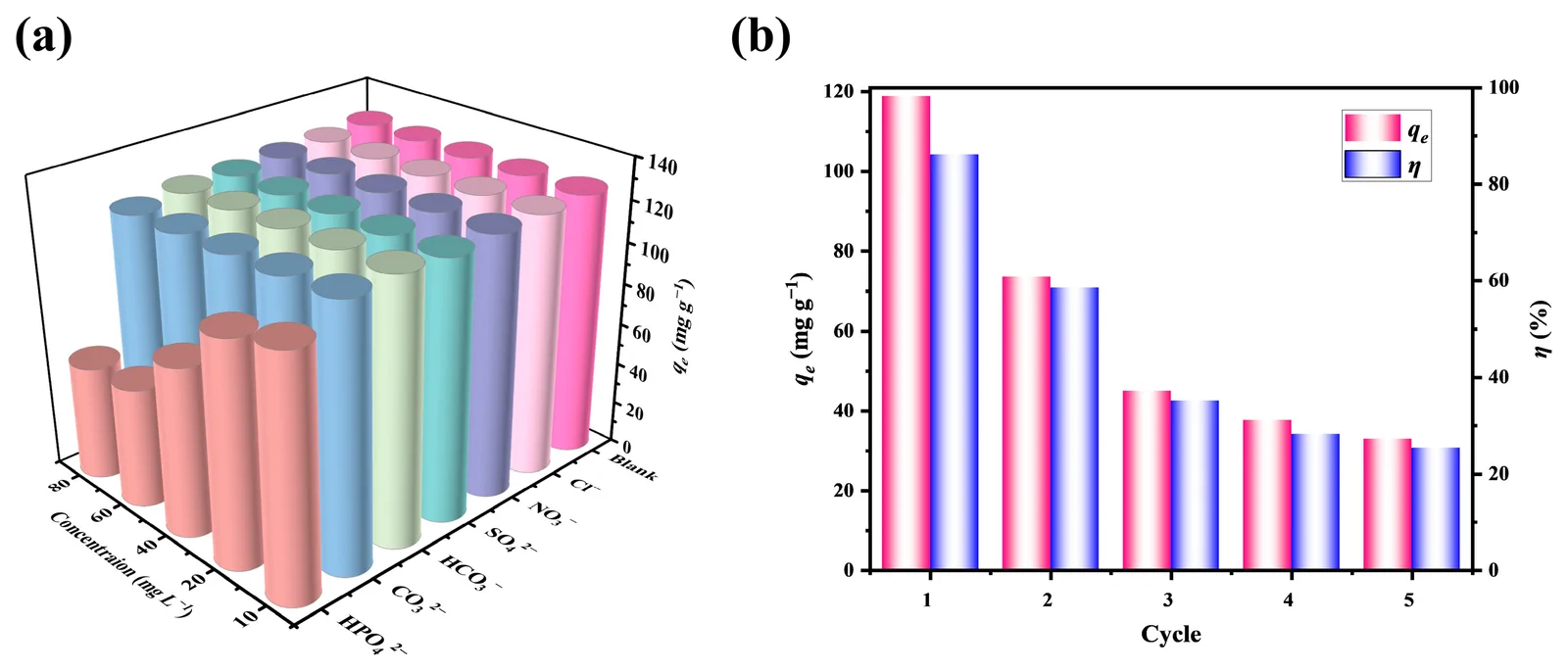
(**a**) Effect of co-existing ions on adsorption performance; (**b**) recycling performance.

In practical applications, aquatic environments are highly complex, and coexisting ions or organic substances in water may influence the removal efficiency of F^−^. Therefore, investigating the effects of coexisting ions is necessary. To evaluate the influence of common coexisting anions on the adsorption performance of Ce–Al–La–MOFs@PPy toward F^−^ in real water systems, the interference effects of HPO_4_^2−^, CO_3_^2−^, HCO_3_^−^, SO_4_^2−^, NO_3_^−^, and Cl^−^ were investigated, as shown in Figure 7a. Compared with the blank system, all coexisting anions exerted certain inhibitory effects on the adsorption process; however, the extent of inhibition varied significantly among different ions. Among them, HPO_4_^2−^ exhibited the most pronounced suppression effect. An increase in the concentration from 10 to 80 mg·L^−1^ resulted in a significant decline in *q_e_*, falling from 113.44 to 54.93 mg·g^−1^. In contrast, CO_3_^2−^, HCO_3_^−^, SO_4_^2−^, NO_3_^−^, and Cl^−^ showed relatively minor effects on adsorption performance, with *q_e_* values generally remaining within the range of 119–125 mg·g^−1^. This phenomenon is mainly attributed to differences in charge density, ionic radius, and coordination ability among various anions [40]. HPO_4_^2−^ possesses a relatively high charge density and strong coordination ability, enabling it to preferentially form inner-sphere coordination interactions with metal active sites such as Ce^3+^ and La^3+^, thereby occupying adsorption sites and significantly suppressing the adsorption of F^−^. In addition, its strong complexation ability may alter the local surface charge environment of the adsorbent, further reducing adsorption efficiency. In comparison, although CO_3_^2−^ and HCO_3_^−^ belong to the carbonate system, their interactions with metal active sites are relatively weaker and mainly influence adsorption through competitive electrostatic interactions. Meanwhile, SO_4_^2−^, NO_3_^−^, and Cl^−^ primarily participate through outer-sphere coordination or electrostatic competition. Owing to their relatively weak coordination abilities, their occupation of adsorption sites is limited, resulting in only minor effects on adsorption performance. This trend is consistent with previous studies showing that multivalent anions, especially those with strong coordination abilities such as HPO_4_^2−^, exhibit significantly stronger interference effects on adsorption systems than monovalent anions [8].

### 3.6. Regeneration Performance Evaluation

To evaluate the regeneration performance of Ce–Al–La–MOFs@PPy, the fluoride-saturated adsorbent was desorbed using NaOH solution, followed by cyclic adsorption–regeneration experiments. The results indicate that both *q_e_* and *η* gradually decreased with increasing regeneration cycles (Figure 7b). Specifically, during the first cycle, the adsorption capacity (*q_e_*) and removal efficiency (*η*) were 118.83 mg·g^−1^ and 86.16%, respectively, while after five cycles they decreased to 32.96 mg·g^−1^ and 25.36%, respectively. Among the regeneration cycles, the adsorption performance decreased more significantly during the first two cycles, whereas the decline became relatively moderate in subsequent cycles. This phenomenon is mainly attributed to the fact that some coordination bonds formed between F^−^ ions and metal active sites such as Ce and La cannot be completely dissociated by NaOH during the adsorption–desorption process, resulting in irreversible loss of active sites [41]. In addition, the alkaline regeneration conditions may partially damage the MOFs or induce hydrolysis of organic ligands, thereby reducing the structural stability and specific surface area of the material [17]. Nevertheless, the material still retained a certain adsorption capacity after five regeneration cycles, indicating that Ce–Al–La–MOFs@PPy possesses potential for regeneration and reuse.

### 3.7. Practical Application Study

To further verify the application potential of Ce–Al–La–MOFs@PPy in complex real-water systems, equipment-cleaning wastewater collected from a chemical enterprise in the Shaowu Industrial Park, Fujian Province, was selected for treatment evaluation. As shown in Table 5, the wastewater was weakly acidic (pH = 3.68) and contained relatively high concentrations of inorganic ions (such as Cl^−^, SO_4_^2−^, and NO_3_^−^) as well as a certain amount of organic pollutants (COD = 126.5 mg·L^−1^), exhibiting the typical characteristics of industrial cleaning wastewater. After treatment with Ce–Al–La–MOFs@PPy, the fluoride concentration decreased significantly from 12.34 mg·L^−1^ to 0.81 mg·L^−1^, corresponding to a removal efficiency exceeding 93%. This result demonstrates that the material still maintains excellent selective fluoride removal capability under complex water-quality conditions. Furthermore, the residual fluoride concentration in the treated effluent complied with the regulatory limits set by the Integrated Wastewater Discharge Standard (GB 8978–1996, ≤10 mg·L^−1^) and the Standards for Drinking Water Quality (GB 5749–2022, ≤1.0 mg·L^−1^). Conversely, the concentrations of coexisting anions (Cl^−^, NO_3_^−^, SO_4_^2−^) exhibited negligible reductions, suggesting that these background ions had a minimal inhibitory effect on the adsorption performance. This further confirms the preferential selective adsorption behavior of the material toward F^−^. Meanwhile, the COD value decreased from 126.5 mg·L^−1^ to 64.3 mg·L^−1^ after adsorption treatment. This decrease suggests that the adsorbent may exhibit a certain capacity for reducing organic pollutants during fluoride removal. However, the specific contribution of PPy and the porous MOF structure to organic pollutant removal remains unclear and requires further investigation through systematic organic compound identification and adsorption studies. Overall, Ce–Al–La–MOFs@PPy exhibits strong anti-interference capability against coexisting ions and stable fluoride removal performance in practical industrial wastewater systems, while simultaneously demonstrating partial organic pollutant removal capability. These results highlight its promising application potential for the treatment of fluoride-containing industrial wastewater.

It should be noted that the practical applicability of Ce–Al–La–MOFs@PPy should be assessed not only by fluoride removal efficiency but also by cost, dosage, secondary pollution, and post-treatment requirements. Conventional salts such as AlCl_3_, FeCl_3_, and LaCl_3_ are inexpensive and can remove fluoride through precipitation, hydrolysis, coagulation, and complexation, but they may introduce residual metal ions and chloride ions, require pH adjustment, and generate chemical sludge. In contrast, Ce–Al–La–MOFs@PPy acts as an insoluble solid adsorbent, with fluoride removal mainly occurring through surface adsorption and coordination exchange with Ce/La active sites. Moreover, the material showed good fluoride selectivity in the presence of coexisting ions and partial removal of organic pollutants in real industrial wastewater. Therefore, this material may be more suitable for selective fluoride polishing or advanced treatment rather than direct replacement of low-cost precipitation agents. Further column tests, regeneration optimization, stability evaluation, and techno-economic analysis are still needed to assess its practical feasibility.

### 3.8. Fluoride Removal Mechanism Analysis

**Figure 8:**
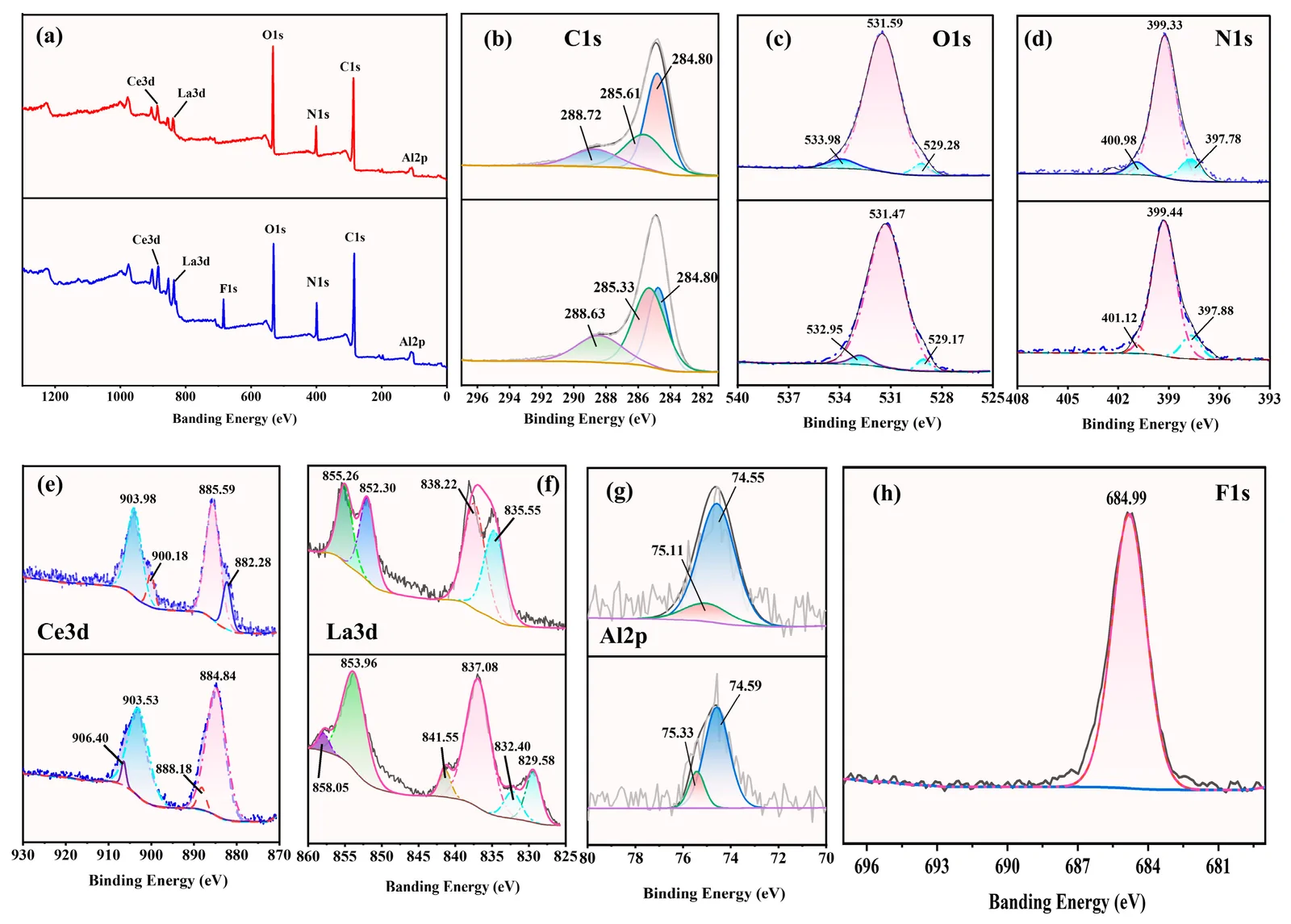
XPS spectra of Ce–Al–La–MOFs@PPy before and after F^−^ adsorption: (**a**) survey spectrum; (**b**) C1s; (**c**) O1s; (**d**) N1s; (**e**) Ce3d; (**f**) La3d; (**g**) Al2p; (**h**) F1s. (The different colors of the curves are used to distinguish between the raw experimental data (black dots/lines) and the fitted curves (colored lines), as well as to differentiate individual peaks during the deconvolution process.).

Figure 8 displays the XPS survey spectra of Ce–Al–La–MOFs@PPy prior to and following fluoride adsorption. From the survey spectra, it can be observed that the pristine Ce–Al–La–MOFs@PPy mainly consists of C, O, N, Ce, La, and Al elements. Among them, the C1s,O1s and N1s signals originate from the PPy conductive polymer and organic ligand framework, respectively, while the Ce3d, La3d, and Al2p signals correspond to the coordination structures of multimetallic nodes, indicating the successful construction of the composite MOFs. After fluoride adsorption, a distinct F1s characteristic peak appeared at approximately 684.99 eV, confirming that F^−^ ions were effectively immobilized on the material surface or within the pore channels.

For the high-resolution C 1s spectrum, the spectrum before fluoride adsorption was deconvoluted into three characteristic components centered at approximately 284.80, 285.61, and 288.72 eV (Figure 8b). The dominant peak at 284.80 eV was assigned to sp^2^-hybridized carbon, while the peaks at 285.61 and 288.72 eV were attributed to C–O/C–N and O–C=O species, respectively [42]. After fluoride adsorption, the three C 1s components were located at 284.80, 285.33, and 288.63 eV. The relatively small changes in their binding energies compared with those before adsorption suggest that no significant redistribution of the electron density occurred around these carbon species. The C 1s results therefore indicate that the PPy component and organic-ligand framework were not the primary binding sites for fluoride during the adsorption process. For the high-resolution O 1s spectrum (Figure 8c), the peaks centered at 529.28, 531.59, and 533.98 eV before adsorption were assigned to three oxygen-containing species, namely C–O–C, M–O, and O–C=O, respectively [43]. After fluoride adsorption, shifts in the binding energies of these components were observed, indicating that the surface hydroxyl groups directly participated in fluoride capture. Combined with the complete disappearance of the characteristic M–OH vibration band after adsorption in the FTIR spectrum, these results suggest that fluoride adsorption involved hydroxyl substitution or ligand exchange between F^−^ and the metal sites. The high-resolution N 1s spectrum revealed three chemically distinct nitrogen species. The peaks at 397.78, 399.33, and 400.98 eV were assigned to imine-like C=N species, secondary-amine N–H species in the pyrrole rings, and positively charged –NH^+^ species, respectively [44]. After fluoride adsorption, these components shifted slightly to 397.88, 399.44, and 401.12 eV, respectively. These small positive shifts suggest that PPy acted as an auxiliary component that facilitated the enrichment of fluoride ions near the composite surface, thereby promoting their subsequent interaction with the metal active sites. Before adsorption, the Ce3d spectrum consisted of characteristic peaks corresponding to both Ce^4+^ and Ce^3+^ valence states. The strong peaks located at 885.59 eV and 903.98 eV were attributed to the main 3d_5_/_2_ and 3d_3_/_2_ peaks of Ce^4+^, respectively, while additional sub-peaks associated with Ce^3+^ could be observed near 882.28 eV and 900.18 eV. These results indicate the presence of mixed Ce^3+^/Ce^4+^ valence states within Ce–Al–La–MOFs@PPy [14,45], which is beneficial for providing variable-valence active sites. The changes observed in the La3d region were even more pronounced. After adsorption, the peaks shifted overall toward higher binding energies and new sub-peaks appeared. This behavior is mainly attributed to the formation of La–F coordination bonds between fluoride ions and La active sites. The strong electronegativity of F^−^ reduces the electron cloud density around La atoms, thereby increasing the binding energy [37]. For the high-resolution Al 2p spectrum, the Al 2p_3_/_2_ component located at approximately 74.55 eV was assigned to the Al–O coordination structure within the framework, while the corresponding Al 2p_1_/_2_ component appeared at approximately 75.11 eV due to spin–orbit splitting. After fluoride adsorption, the Al 2p doublet remained at similar binding-energy positions, indicating that no significant change occurred in the oxidation state of Al during adsorption. Because Al^3+^ possesses strong Lewis acidity, the exposed Al–O active sites can interact with F^−^ through Lewis acid–base interactions and surface ligand exchange. The Al centers in Ce–Al–La–MOFs@PPy may therefore serve as auxiliary adsorption sites and participate in fluoride capture together with the Ce/La-based oxygen-coordinated centers. Therefore, the fluoride adsorption behavior of Ce–Al–La–MOFs@PPy is primarily attributed to the synergistic coordination adsorption effects of multimetallic nodes, particularly Ce and La, which collectively promote the adsorption and efficient removal of F^−^ on the material surface.

SEM images demonstrate that Ce–Al–La–MOFs@PPy possesses a flower-like structure with hierarchical pores, providing abundant active adsorption sites. The N_2_ adsorption–desorption isotherms further confirm that the material exhibits a mesoporous-dominated porous structure. Combined with XPS, FTIR, XRD, adsorption kinetics, and thermodynamic analyses, the fluoride adsorption mechanism was systematically elucidated. Thermodynamic results indicate that both physical adsorption and chemical interactions coexist during the adsorption process, while the kinetic and isotherm analyses suggest that fluoride uptake is predominantly governed by monolayer chemisorption.

**Figure 9:**
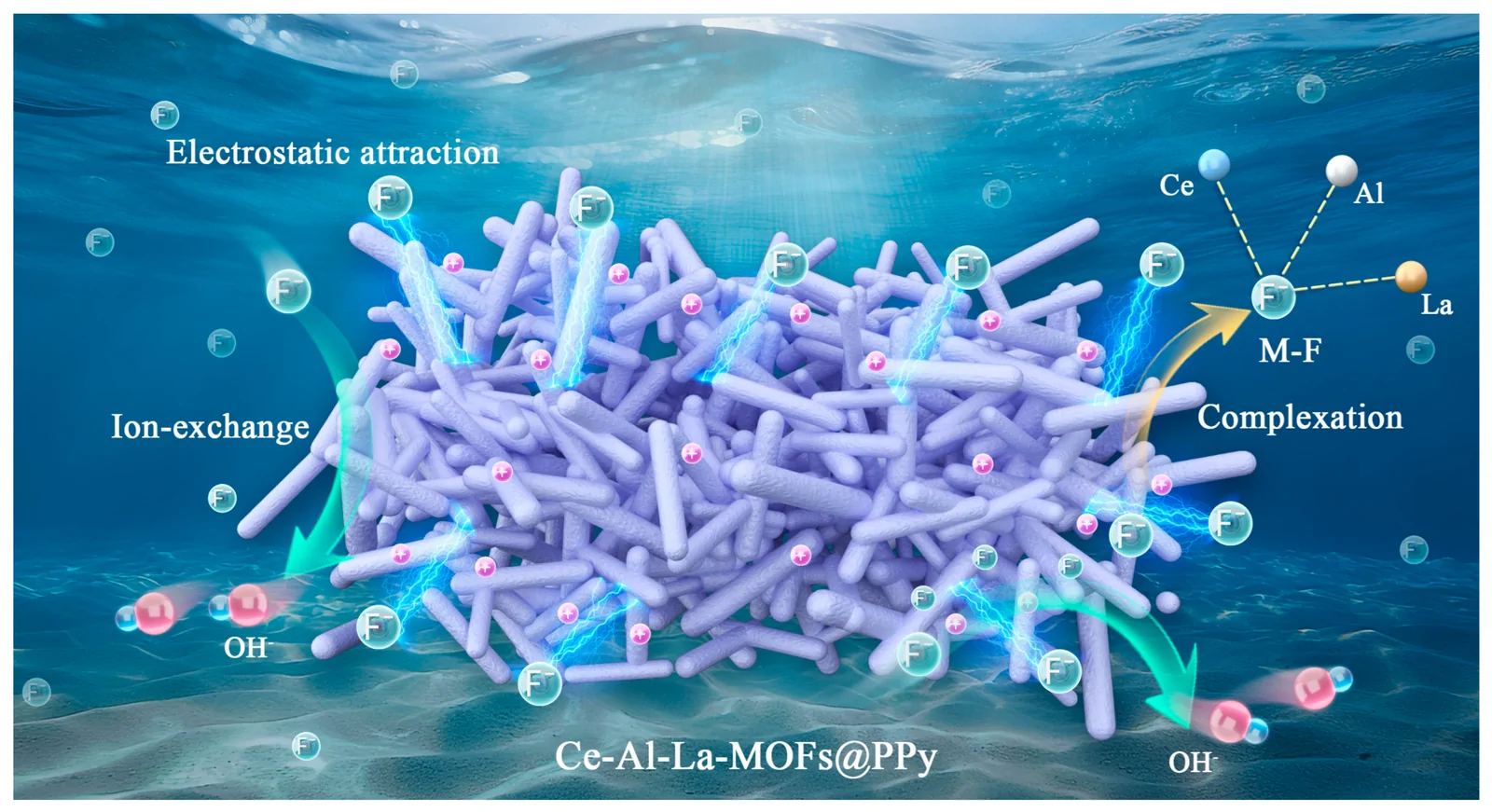
Mechanism scheme of fluoride removal by Ce–Al–La–MOFs@PPy.

In the present system, PPy acts primarily as a nitrogen-rich interfacial adsorption layer. Protonated or oxidatively doped nitrogen sites in PPy create positively charged regions that promote the interfacial enrichment of F^−^ through electrostatic attraction. Pyrrolic N–H groups may provide additional N–H···F interactions. These processes increase the local fluoride concentration near the MOF surface and facilitate its subsequent transfer to the adjacent metal active sites. Owing to the strong Lewis acidity of Ce and La active sites, F^−^ ions are first enriched near the adsorbent surface through electrostatic attraction and subsequently replace surface hydroxyl groups via ligand exchange. The coordinated fluoride ions further form stable inner-sphere metal–fluoride complexes (Ce–F and La–F). At locally high fluoride concentrations, part of the coordinated fluoride may further evolve into sparingly soluble rare-earth fluoride species (e.g., CeF_3_ and LaF_3_) on the adsorbent surface, thereby enhancing fluoride immobilization. Therefore, fluoride removal by Ce–Al–La–MOFs@PPy is achieved through the synergistic effects of electrostatic attraction, ligand exchange, surface complexation, and localized rare-earth fluoride precipitation (Figure 9).

(3) $$M-OH(s)+F^{-}\to M-F(s)+OH^{-}$$

(4) $$M-O{H_{2}}^{+}(s)+F^{-}\to M-F(s)+H_{2}O$$

### 3.9. Performance Assessment

**Figure 10:**
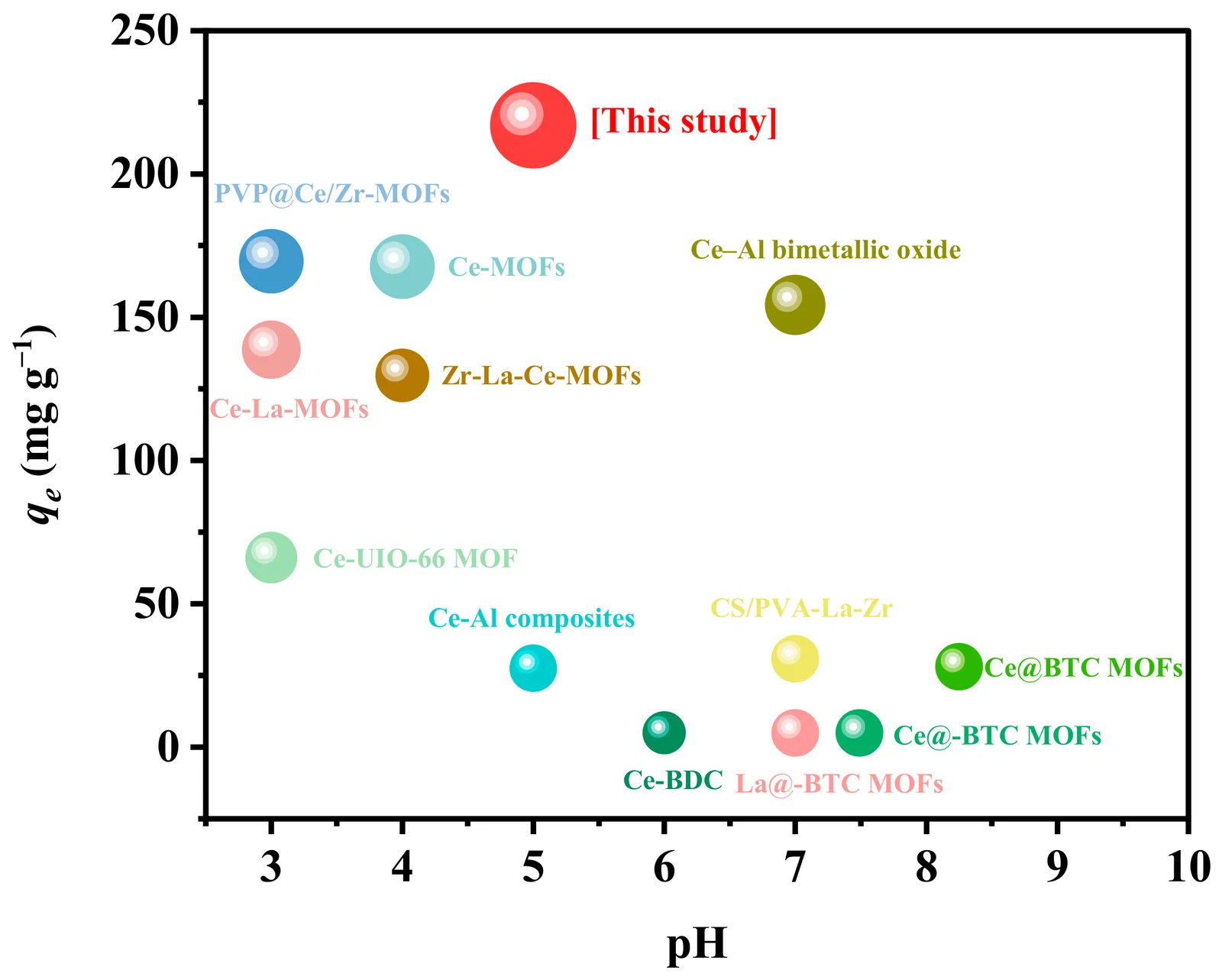
Performance comparison of various adsorbents reported in previous and Ce–Al–La-MOFs@PPy.

Table 6 and Figure 10 compare the fluoride adsorption performance of Ce–Al–La–MOFs@PPy with those of previously reported adsorbent materials. Significant differences in fluoride removal performance can be observed among various adsorbents. Traditional single-component MOFs materials, such as Ce-BDC and Ce@BTC MOFs, generally exhibit relatively low adsorption capacities, mostly around 5 mg·g^−1^. In contrast, some bimetallic or modified materials, including Ce-La-MOFs and Ce–Al bimetallic oxides, show improved adsorption performance, with adsorption capacities reaching approximately 100–150 mg·g^−1^. Furthermore, through polymer compositing or multimetal synergistic modification, such as in PVP@Ce/Zr-MOFs and trimetallic Zr–La–Ce MOFs, the adsorption capacity can be further increased to above 160 mg·g^−1^, indicating that structural regulation and interfacial modification play important roles in enhancing adsorption performance. Compared with these reported materials, the Ce–Al–La–MOFs@PPy constructed in this study achieved a maximum fluoride adsorption capacity of 216.92 mg·g^−1^ under conditions of pH = 5 and 45 °C, which is significantly higher than that of most previously reported adsorbents, demonstrating excellent fluoride removal capability. These results indicate that the synergistic effect of the trimetallic system combined with conductive PPy polymers not only increases the number of active sites, but also optimizes the surface charge properties and pore structure, thereby significantly enhancing the adsorption performance toward F^−^. Consequently, the material exhibits strong application potential and competitive advantages in fluoride removal.

## 4. Conclusions

Polypyrrole-modified Ce–Al–La trimetallic metal–organic framework composite adsorbent was successfully synthesized through a solvothermal method combined with in situ polymerization. By regulating the molar ratio between metal species and organic ligands, the optimal synthesis condition was determined to be a metal/ligand ratio of 3:1. Under this condition, the material exhibited a flower-like hierarchical structure assembled from nanorods, possessing abundant pores and uniformly distributed multimetal active sites. Structural characterization results (SEM, XRD, FTIR, BET, and XPS) confirmed that PPy was successfully integrated into the MOFs, which not only improved the microstructure of the material but also provided a favorable interfacial environment for subsequent adsorption reactions. Adsorption performance studies demonstrated that Ce–Al–La–MOFs@PPy possesses excellent fluoride removal capability. The adsorbent demonstrated peak performance at pH 5, with the uptake capacity exhibiting a positive dependence on the initial fluoride concentration. The adsorption isotherms conformed to the Langmuir model, signifying monolayer coverage on a homogeneous surface, achieving a theoretical maximum (*Q_m_*) of 216.92 mg·g^−1^. Kinetic analysis revealed that the process followed the PSO model, indicating that chemisorption was the rate-limiting step. Furthermore, thermodynamic evaluations confirmed the spontaneity (Δ*G°* < 0) and endothermic nature (Δ*H°* > 0) of the adsorption, which was also entropy-driven (Δ*S°* > 0). Mechanistic investigations demonstrated that the elimination of fluoride by Ce–Al–La–MOFs@PPy mainly originated from electrostatic interactions and coordination-exchange mechanisms, in which Ce and La served as the primary active sites. The material showed good resistance to most common coexisting anions, whereas phosphate species, particularly HPO_4_^2−^, caused significant inhibition of fluoride adsorption. Meanwhile, Ce–Al–La–MOFs@PPy exhibited certain regeneration potential. Overall, this multimetallic MOFs@PPy composite material shows significant advantages in structural design and performance regulation, highlighting its promising application prospects in fluoride-containing wastewater treatment. However, further studies on metal leaching, toxicity, long-term stability, column operation, and regeneration optimization are still required before practical application.

## Figures and Tables

**Table 1:** Langmuir and Freundlich isotherm model fitting parameters.

°C	Langmuir			Freundlich		
	*Q_m_* (mg·g^−1^)	*K_L_* (L mg^−1^)	*R* ^2^	*K_f_* (L g^−1^)	*n*	*R* ^2^
25	170.3578	1.1465	0.9994	106.4633	6.7981	0.7237
35	204.0816	0.9533	0.9983	123.2452	6.7173	0.7465
45	216.9197	0.8882	0.9987	125.3978	6.1166	0.7887

**Table 2:** Thermodynamic parameters for F^−^ adsorption on Ce–Al–La–MOFs@PPy at different temperatures.

*T* (K)	Δ*G*° (kJ/mol)	Δ*H*° (kJ/mol)	Δ*S*° (J/mol·K)
298	−4.1223	9.3330	45.1522
308	−4.5738		
318	−5.0253		

**Table 3:** Fitting parameters of PFO and PSO adsorption kinetic models.

Model	Concentration (mg/L)	k	*q_e_* (mg·g^−1^)	*R* ^2^
PFO	10	0.0125	15.5304	0.9020
	20	0.0098	43.1508	0.9308
PSO	10	0.0020	93.0233	0.9999
	20	0.0005	136.7989	0.9996

**Table 4:** Fitting parameters of the intraparticle diffusion model.

Concentration (mg/L)	Equation		*R* ^2^
10	Stage I	*q_t_* = 29.1855 + 7.9740 *t*^0.5^	0.8980
	Stage II	*q_t_* = 83.8621 + 0.4656 *t*^0.5^	0.9147
	Stage III	*q_t_* = 91.9800 + 0.0033 *t*^0.5^	0.1757
20	Stage I	*q_t_ =* −5.5246 + 15.0173 *t*^0.5^	0.9959
	Stage II	*q_t_* = 111.3844 + 1.1204 *t*^0.5^	0.8933
	Stage III	*q_t_* = 132.4952 + 0.0114 *t*^0.5^	0.0500

**Table 5:** Actual wastewater quality parameters.

Parameters	Before	After
F^−^ concentration (mg·L^−1^)	12.34	0.81
pH	3.68	4.92
NH_3_-N (mg·L^−1^)	3.12	2.95
Cl^−^ (mg·L^−1^)	48.6	46.2
CO_3_^2−^ (mg·L^−1^)	ND	ND
NO_3_^−^ (mg·L^−1^)	6.85	6.42
SO_4_^2−^ (mg·L^−1^)	58.3	52.7
COD (mg·L^−1^)	126.5	64.3

**Table 6:** Comparison of defluorination performance of Ce–Al–La–MOFs@PPy with previously reported adsorbent materials.

Adsorbents	pH	*q_m_* (mg·g^−1^)	Reference
Ce-UIO-66 MOF	3	66.1	[46]
Ce-BDC	6–7	4.91	[41]
Ce-MOF	4	129.7 (50 °C)	[11]
Ce@ BTC MOFs	7.49	4.94 (50 °C)	[13]
La@ BTC MOFs	7.22	4.98 (30 °C)	[13]
La@ ABDC MOF	7	4.95 (30 °C)	[47]
Ce-Al composites	5	27.5	[48]
Ce-La-MOFs	3	138.64 (50 °C)	[14]
Ce–Al bimetallic oxide	7	154.28 (50 °C)	[49]
CS/PVA-La-Zr	7	30.73	[50]
PVP@Ce/Zr-MOFs	3	169.49 (45 °C)	[45]
La-Al-Fe	8.25	28.06 (25 °C)	[51]
Tri-metallic Zr-La-Ce MOFs	4	167.61 (45 °C)	[15]
Ce–Al–La–MOFs@PPy	5	216.92 (45 °C)	This study

## Data Availability

The original contributions presented in this study are included in the article and Supplementary Material. Further inquiries can be directed to the corresponding authors.

## Supplementary Materials

The following supporting information can be downloaded at: https://www.mdpi.com/article/10.3390/nano16161015/s1. Text S1. Adsorption thermodynamics; Text S2. Adsorption kinetics; Figure S1: Comparison of the fluoride adsorption capacities of PPy, Ce–Al–La–MOFs, and Ce–Al–La–MOFs@PPy.
