# Supplementary material for: Construction of Hierarchical Rod-Assembled Ce–Al–La–MOFs@PPy Composites for Highly Efficient Fluoride Removal from Water
Source: Nanomaterials (Basel). 2026 Aug 17;16(16):1015. doi: 10.3390/nano16161015 (PMC13515641; doi:10.3390/nano16161015)
Supplement: Supplementary file 1 [file nanomaterials-16-01015-s001.zip › nanomaterials-4412866-supplementary.pdf]

Supplementary Information

Construction of Hierarchical Rod-Assembled Ce–Al–La–MOFs@PPy Composites for Highly Efficient Fluoride Removal from Water

Xin Lin [1,2], Jiayi Tu [1,3], Jinyun Zhao [1], Fangfang Wu [1,*], Xingping Fu [1,*], Hao Lin [1,3,*] and Jiapeng Hu [1]

1 Fujian Provincial Key Laboratory of Eco-Industrial Green Technology, Wuyi University, Wuyishan 354300, China; linxin820314@163.com (X.L.); tujiayi@163.com (J.T.); zhaojy@163.com (J.Z.); wyuwqhjp@163.com (J.H.)

2 Fujian Environmental Protection Design Institute Company Limited, Fuzhou 350001, China

3 College of Resources and Environment, Fujian Agriculture and Forestry University, Fuzhou 350002, China

* Correspondence: wuff2018@wuyiu.edu.cn (F.W.); fuxingping0224@126.com (X.F.); linhaosg@wuyiu.edu.cn (H.L.)

Text S1. Adsorption thermodynamics

The Langmuir isotherm model can be expressed by the following Equation:

$$C_e / q_e = 1 / K_L q_m + C_e / q_m \quad \text{(S1)}$$

The Freundlich isotherm model equation is expressed as follows:

$$\lg q_e = \lg K_f + \frac{1}{n} \lg C_e \quad \text{(S2)}$$

Herein: q_e (mg g^{-1}): the equilibrium adsorption capacity; q_m (mg g^{-1}): the maximum adsorption capacity; C_e (mg L^{-1}): the equilibrium F^- concentrations, K_L (L mg^{-1}): the adsorption equilibrium constant; K_f (L·g^{-1}): the empirical constants of Freundlich isotherm; n is the adsorption constant reflecting the adsorption strength.

K_d can be calculated by Equation (S3):

$$K_d = q_e / C_e \quad \text{(S3)}$$

The *van't Hoff* equation is expressed by Equation (S4):

$$\ln K_d = -\Delta H^o / RT + \Delta S^o / R \quad \text{(S4)}$$

Gibbs free energy (ΔG^o) can be calculated by Equations (S5):

$$\Delta G^o = \Delta H^o - T \times \Delta S^o \quad \text{(S5)}$$

Where the ΔG^o (kJ mol^{-1}): the free energy change; ΔH^o (kJ mol^{-1}): the enthalpy change; ΔS^o (J mol^{-1} K^{-1}): the entropy change; R: the molar gas constant, 8.314 J mol^{-1} K^{-1}; K_d: the distribution coefficient; T (K): the adsorption reaction temperature. The thermodynamic parameters were estimated using the commonly adopted distribution coefficient $K_d = q_e/C_e$, with q_e and C_e expressed in mg·g^{-1} and mg·L^{-1}, respectively. It should be noted that the resulting K_d is unit-dependent rather than a rigorous dimensionless standard equilibrium constant. Therefore, the calculated values are interpreted as apparent thermodynamic parameters and are used primarily to describe the temperature-dependent adsorption trend under the fixed unit convention employed in this study. This limitation does not affect the comparative conclusion that fluoride adsorption becomes more favorable with increasing temperature.

Text S2. Adsorption kinetics

Pseudo-first order model:

$$\ln(q_e - q_t) = \ln q_e - k_1 t \qquad \text{(S6)}$$

Pseudo-second order model:

$$\frac{t}{q_t} = \frac{1}{k_2 q_e^2} + \frac{t}{q_e} \qquad \text{(S7)}$$

Intra-particle diffusion model:

$$q_t = k_p t^{\frac{1}{2}} + C \qquad \text{(S8)}$$

In the above expressions, q_t (mg g^{-1}): the amount of fluoride adsorbed at time t; q_e (mg g^{-1}): the amount of fluoride adsorbed at equilibrium; k_1 (min^{-1}): the pseudo-first-order adsorption rate constant; k_2 (g min^{-1} mg^{-1}): the pseudo-second-order adsorption rate constant; k_p (mg min$^{-1/2}$ g^{-1}): the intra-particle diffusion rate constant; C denotes the intercept providing an idea of the boundary layer thickness.

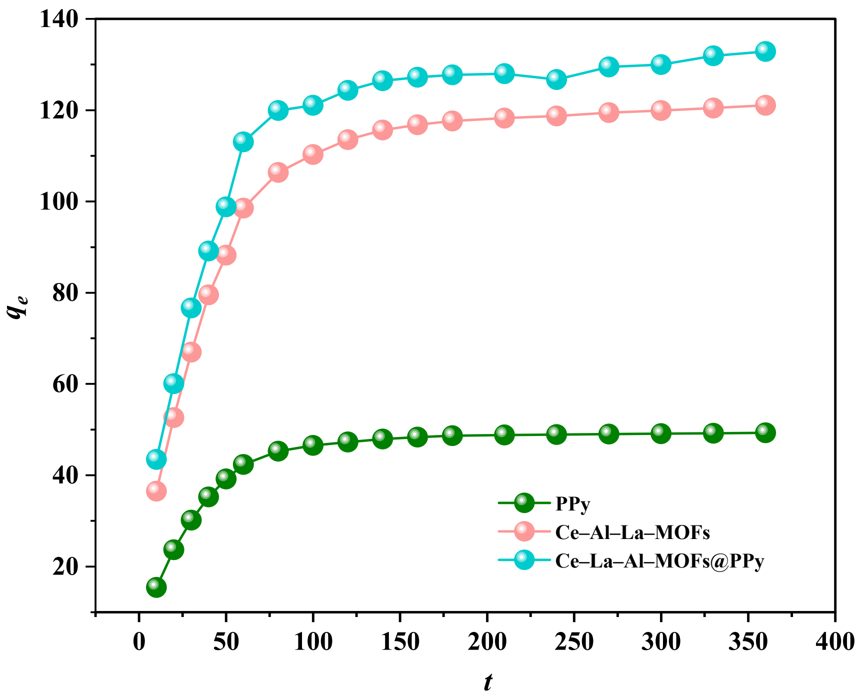

Figure S1. Comparison of the fluoride adsorption capacities of PPy, Ce–Al–La–MOFs, and Ce–Al–La–MOFs@PPy.
